# A multidimensional Elo method for online item calibration

**DOI:** 10.3389/fpsyg.2026.1849155

**Published:** 2026-10-09

**Authors:** Jiahui Zhang, Yuqing Yuan, Fuhang Zhang, Feng Li

**Affiliations:** 1 Collaborative Innovation Center of Assessment for Basic Education Quality, Beijing Normal University, Beijing, China; 2 School of Statistics, Beijing Normal University, Beijing, China; 3 Collaborative Innovation Center of Assessment for Basic Education Quality, Beijing Normal University, Zhuhai, China; 4 College of Education for the Future, Beijing Normal University, Zhuhai, China

**Keywords:** CAT, Monte Carlo simulation, multidimensional ability estimation, multidimensional Elo, multidimensional IRT, online item calibration

## Abstract

**Introduction:**

As adaptive testing and learning systems proliferate, efficient item calibration is a pressing need. The Elo rating system jointly estimates student ability and item difficulty, offering an alternative to item response theory (IRT) models. Yet existing multidimensional extensions have mainly focused on ability tracking rather than item calibration.

**Methods:**

This study proposes the multidimensional Elo (MELO) method for online item calibration when no calibrated item pool or anchor items are available. Two simulations (500 replications per condition) evaluated accuracy via RMSE and conditional bias. Study 1 compared MELO with multidimensional IRT (MIRT) estimation across dimensional structures (between-item vs. within-item), inter-dimensional correlations, and sample sizes. Study 2 manipulated dimensional structure, adaptive selection proportion, and sample size–test length combinations at a fixed number of responses. An empirical application compared MELO and MIRT item difficulty estimates using real response data.

**Results:**

For item difficulty, the accuracy gap between MELO and MIRT was modest and largely confined to extremely difficult items; under within-item multidimensionality with strong inter-dimensional correlation, MELO was significantly more accurate. Conditional bias exhibited a regression-to-the-mean pattern concentrated at the distribution extremes. Adaptivity generally benefited within-item multidimensionality, whereas under between-item structures only partial adaptivity following a random item-selection phase was beneficial, and fully adaptive selection induced substantial item exposure imbalance and degraded calibration. The effect of the sample size–test length configuration on item calibration was moderated by dimensional structure and adaptive selection proportion. Longer tests administered to fewer examinees improved ability estimation. In the empirical data, MELO and MIRT item difficulty estimates correlated almost perfectly (*r* = 0.997).

**Discussion:**

The findings support MELO for preliminary item calibration under the conditions examined.

## 1 Introduction

The rapid advancement of e-learning technologies has transformed modern education, with computerized adaptive testing (CAT) and intelligent tutoring systems (ITSs) becoming increasingly prevalent (He and Chen, 2020). More recently, artificial intelligence (AI)-driven item generation has begun to produce candidate items at a pace that conventional calibration pipelines cannot match: when items can be generated automatically and continuously, item parameters must be estimated with comparable speed (Graesser et al., 2018). The effectiveness of these adaptive systems fundamentally depends on the quality of their underlying item pools and, in particular, on the accuracy of item parameters. Item calibration, the process of estimating item parameters (Baker and Kim, 2004), has thus become a critical technical component supporting personalized learning. However, traditional calibration methods based on item response theory (IRT) rely on large-scale pretesting data, making them expensive and severely constraining their timeliness and flexibility (Embretson and Reise, 2013; van der Linden and Hambleton, 1997). Consequently, researchers have proposed adapting the Elo rating system (ERS) as a more agile alternative to IRT-based calibration.

Originally developed as a dynamic rating algorithm for chess players (Elo, 1978), the ERS has gained attention in educational assessment for its computational simplicity, ease of implementation, and independence from strict distributional assumptions and large-sample requirements (Antal, 2013; Pelánek, 2016; Wauters et al., 2010). In this framework, test taking is conceptualized as a “game” between the student and the item, and student ability and item difficulty are updated simultaneously after each response. Elo-type updating thus offers a promising basis for item calibration, because item and person parameters are refined as responses accumulate, without requiring large calibration samples or iterative batch estimation (Vermeiren et al., 2026). Empirical studies have demonstrated that the ERS provides adequate estimation precision across different learner populations and subjects, and the ERS achieved performance comparable to IRT under both random and adaptive item selection, whereas proportion-correct approaches are inappropriate under adaptive selection (Pelánek et al., 2017; Vermeiren et al., 2026; Wauters et al., 2012).

The standard ERS, however, rests on a unidimensional ability assumption, that all items measure a single latent trait. This assumption fundamentally misaligns with the realities of learning, in which multiple ability dimensions are typically intertwined. Extending the Elo rating system to accommodate multidimensionality is therefore essential for characterizing both the multidimensional attributes of assessment items and students' multidimensional ability structures. Two distinct dimensional structures must be distinguished: within-item multidimensionality, in which a single item measures multiple dimensions simultaneously, and between-item multidimensionality, in which each item assesses only one dimension (Reckase, 1985, 2009). In addition, multidimensional extensions of the ERS can be divided into fixed low-rank formulations, in which a small number of dimensions is specified in advance (conventional in multidimensional IRT), and high-dimensional variants that permit large or inferentially determined ability spaces (common in adaptive learning contexts). The present study focuses on the former.

To date, two multidimensional Elo systems have been proposed, both developed primarily for ability tracking rather than item calibration. Park et al. (2019) integrated compensatory multidimensional IRT (Reckase, 2009) into the Elo framework and proposed the Multidimensional Elo Rating System (M-ERS), whose updating rule incorporates a dimensional weight indicator that takes a value of 1 when the administered item assesses a given dimension and a value within [0, 1] otherwise, so that all ability estimates are updated after each item administration. Their simulation studies, however, employed true item parameters without conducting item calibration, because the objective was to monitor examinees' ability progression rather than to estimate item characteristics. Doebler et al. (2015) developed the Multidimensional Elo Tracking Algorithm (META) for between-item structures, extending the standard updating formula with inter-dimensional correlation parameters and an uncertainty function for dimensions not assessed by the current item, together with differential uncertainty functions for ability and item difficulty parameters. Their findings showed that leveraging latent associations between dimensions enhances testing efficiency, but item parameter recovery was not evaluated; the focus remained on the test length required to achieve predetermined precision standards for ability estimation. Whether a multidimensional Elo system can recover item parameters accurately thus remains an open question.

A parallel line of research has developed online calibration procedures within the IRT framework. In unidimensional CAT, sequential procedures such as Method A, the one-EM-cycle (OEM) method, and the multiple-EM (MEM) method have been systematically evaluated (Ban et al., 2001); for multidimensional CAT, Chen et al. (2017) proposed the M-Method A, M-OEM, and M-MEM methods, and Chen and Wang (2016) developed the full-functional MLE-M-Method A, which corrects for estimation error in examinees' ability vectors when calibrating new items. Subsequent work has addressed optimal online calibration designs for item replenishment (He and Chen, 2020), extensions to polytomously scored multidimensional items (Yuan et al., 2023), and neural-network-based approaches that bypass marginal maximum likelihood estimation to alleviate convergence problems in high-dimensional settings (Yuan et al., 2026). Although these likelihood-based procedures achieve accurate item parameter recovery, they share features that constrain their applicability when an online education system is first established: they rely on iterative EM or maximum likelihood estimation at each calibration step, incur substantial computational costs, and presuppose an operational item pool with calibrated anchor items to which new items are linked. These presuppositions do not hold for a newly established system: no calibrated items and no anchor items yet exist, examinee responses accumulate gradually so that early calibration necessarily relies on small samples, and the number of items awaiting calibration is typically large. Preliminary calibration under such conditions is a recurring challenge, because conventional likelihood-based estimation requires relatively large samples and may fail to converge when data are sparse.

Extending ERS to multidimensional contexts presents several unresolved limitations. The models proposed by Park et al. (2019) and Doebler et al. (2015) share a similar structural logic: both apply a decay coefficient (less than 1) to updates for dimensions not directly assessed by the administered item, thereby ensuring that the targeted dimension undergoes substantial updating while related but non-targeted dimensions receive more modest adjustments. Specifically, Park et al. (2019) multiply non-targeted dimensions by a weighting coefficient *D*_*m*(*t*)_ (constrained between 0 and 1), though the selection of this coefficient remains largely arbitrary in their formulation. Conversely, Doebler et al. (2015) employ an alternative uncertainty function, *K*_*other*_, scaled by the inter-dimensional correlation coefficient. The present study integrates these two approaches to define a multidimensional ERS framework coupled with an adaptive item selection strategy, with particular emphasis on evaluating its efficacy as a method for item calibration.

## 2 The present study

The present study addresses this cold-start scenario by proposing the multidimensional Elo (MELO) method, which differs from existing online MIRT calibration procedures in three respects. First, MELO is model-free: item difficulty and examinee ability parameters are updated sequentially after each response via an Elo-type rule, without solving likelihood functions or running EM cycles. Second, rather than calibrating only a small set of new items against a fixed operational pool, MELO jointly calibrates all items alongside ability estimation, making it suitable for *de novo* item pool construction in adaptive practice systems. Third, its per-response computational cost is negligible, enabling real-time calibration at scale. MELO should thus be regarded as a lightweight, complementary alternative to likelihood-based online calibration.

MELO accommodates two multidimensional item-pool structures: between-item multidimensionality, where each item measures only a single dimension, and within-item multidimensionality, where individual items load on multiple ability dimensions. The proposed approach draws on the multidimensional Rasch model (i.e., the multidimensional random coefficients multinomial logit model, MRCMLM; Adams et al., 1997). The Rasch family is widely adopted in online educational applications because of its parsimony, robust estimation properties, and direct interpretability (e.g., Bolsinova et al., 2026). Like the MRCMLM, MELO is compensatory: a deficiency in one ability dimension can be offset by strengths in others.

Let *Y*_*ij*_∈{0, 1} denote examinee *i*'s response to item *j*, and let $\theta_{i}={\left(\theta_{i1},\ldots ,\theta_{iM}\right)}^{⊤}$ denote the examinee's ability vector. The item-by-dimension relationship is represented by a fixed Q matrix, where *q*_*jm*_ = 1 if item *j* measures dimension *m*, and *q*_*jm*_ = 0 otherwise. The MELO response model is a compensatory multidimensional Rasch-type logistic model:


$$\begin{array}{l} p_{ij}=\mathrm{Pr}\left(Y_{ij}=1|\theta_{i},\beta_{j},q_{j}\right)=\frac{\exp\left(\overset{M}{\underset{m=1}{\sum }}q_{jm}\theta_{im}-\beta_{j}\right)}{1+\exp\left(\overset{M}{\underset{m=1}{\sum }}q_{jm}\theta_{im}-\beta_{j}\right)} \end{array}$$ (1)

where β_*j*_ is the difficulty of item *j* and *M* = 3 in the present simulations. Under a between-item multidimensional structure, exactly one element of *q*_*j*_ equals one, so the model reduces to the unidimensional Rasch form for the measured dimension. Under a within-item multidimensional structure, multiple elements of *q*_*j*_ may equal one, allowing abilities on the measured dimensions to compensate for one another. Conditional on the ability vector, item difficulty, and Q matrix, responses are assumed to be conditionally independent in Equation 1.

When examinee *i* responds to item *j* after completing *t* previous items, and item *j* has previously been administered *n* times, the expected probability is computed from the current, pre-update parameter estimates (Equation 2):


$$\begin{array}{l} {\hat{p}}_{ij}^{\left(t,n\right)}=logit^{-1}\left(\overset{M}{\underset{m=1}{\sum }}q_{jm}{\hat{\theta }}_{im}^{\left(t\right)}-{\hat{\beta }}_{j}^{\left(n\right)}\right) \end{array}$$ (2)

After observing response *y*_*ij*_, the ability and difficulty estimates are updated according to Equations 3 and 4, respectively.


$$\begin{array}{l} {\hat{\theta }}_{im}^{\left(t+1\right)}={\hat{\theta }}_{im}^{\left(t\right)}+\left[D+\left(1-D\right)q_{jm}\right]K_{i}\left(t\right)\left(y_{ij}-{\hat{p}}_{ij}^{\left(t,n\right)}\right) \end{array}$$ (3)

$$\begin{array}{l} {\hat{\beta }}_{j}^{\left(n+1\right)}={\hat{\beta }}_{j}^{\left(n\right)}+K_{j}\left(n\right)\left({\hat{p}}_{ij}^{\left(t,n\right)}-y_{ij}\right) \end{array}$$ (4)

where *K*_*i*_(*t*) and *K*_*j*_(*n*) are uncertainty functions that directly determine the system's convergence speed and stability (Glickman, 1999) and the weight *D*∈[0, 1] governs cross-dimensional updating.

Pelánek's (2014) simulation research indicated that updating rules based on hyperbolic forms performed optimally with educational data. Therefore, this study adopted this form to dynamically adjust the updating magnitude when constructing the MELO method. Additionally, considering that in actual systems, items are typically answered far more frequently than individual students, creating significant asymmetry in data structure (Pelánek, 2016), this study used independent uncertainty functions for items and students separately (Equations 5 and 6, respectively).


$$\begin{array}{l} K_{i}\left(t\right)=c_{1}/\left(1+c_{2}t\right) \end{array}$$ (5)

$$\begin{array}{l} K_{j}\left(n\right)=d_{1}/\left(1+d_{2}n\right) \end{array}$$ (6)

An unmeasured dimension receives a weight of *D*, whereas a dimension directly measured by item *j* receives an updating weight of 1. Thus, *D* affects the online updating rule but does not enter the response probability. Both updates use the same expected probability computed before either parameter is updated.

The structure-specific values of *D*, *c*_1_, *c*_2_, *d*_1_, and *d*_2_ are tuning hyperparameters that can be selected through a random search due to the high dimensionality. Because the between-item and within-balanced structures differ in how dimensions load onto items, the optimal learning rates for ability and item updates may differ as well.

Unlike Park et al. (2019) and Doebler et al. (2015) who tied cross-dimensional weights to the latent correlation structure, we did not condition *D* on the data-generating ρ because such correlation information is unavailable before examinee data are collected. MELO is an online calibration algorithm that updates ability and item estimates sequentially during test administration. In a real test session, the true ρ is unknown to the algorithm; the only information available is the Q-matrix. Therefore, conditioning *D* on ρ would violate the online nature of the procedure and is not implementable in practice.

Whether MELO can achieve item calibration performance comparable to multidimensional IRT (MIRT) remains an open empirical question. Existing approaches to online multidimensional calibration have typically focused on ability estimation precision or computational efficiency, with less attention to item-parameter recovery in small-sample pretesting contexts. For instance, Park et al. (2019) derived updating formulas for item difficulty parameters but treated these as known constants in their empirical validation, thereby precluding direct assessment of calibration accuracy. Doebler et al. (2015) incorporated item parameter updating, yet their investigation was restricted to between-item multidimensional structures and emphasized the test length required to achieve predetermined ability estimation precision, without examining how inter-dimensional correlations or sample sizes might jointly affect the recovery of item and ability parameters. Consequently, the relative performance of MELO and MIRT in small-sample settings where item calibration is the primary objective—across different dimensional structures and correlation levels—has yet to be established.

Previous research has demonstrated that unidimensional ERS can achieve performance highly similar to IRT methods under adaptive item selection strategies, whereas IRT is computationally more demanding (Pelánek, 2016). Such systems have been deployed in adaptive learning environments like Math Garden, providing simple and efficient update rules for dynamic parameter calibration (Klinkenberg et al., 2011). However, extending these systems to multidimensional adaptive testing raises practical questions that remain unaddressed. Specifically, when operating under a fixed total response burden that must be allocated across varying sample size and test length combinations, it is unclear how different adaptive selection proportions and dimensional structures influence item exposure patterns and the accuracy of item and ability parameter recovery. Thus, the feasibility and performance characteristics of MELO in multidimensional adaptive calibration systems require further empirical investigation.

This study focuses on the following two research questions:

*Research Question 1: In small-sample pretesting contexts (N* ≤ *250) where item calibration is the primary objective, how does the MELO method compare with MIRT in terms of item-parameter recovery and ability-parameter recovery accuracy (measured by RMSE and bias) across different dimensional structures, inter-dimensional correlations, and sample sizes?*

*Research Question 2: Under a fixed total response burden (N* × *L* = *36,000, yielding an average of 200 responses per item) allocated across different sample size and test length combinations, where adaptive testing is employed to enhance the examinee experience while item calibration remains the primary objective, what are the item-parameter recovery accuracy, item exposure patterns, and ability-parameter recovery accuracy (in terms of RMSE and bias) of the MELO method across different adaptive selection proportions, dimensional structures, and N-by-L configurations?*

## 3 Methods

This study employed simulation experiments to examine the item calibration performance of the proposed MELO method. Study 1 focused on comparing the calibration accuracy of the newly proposed MELO method with the MIRT model; Study 2 further examined calibration accuracy and item exposure under different adaptive item-selection proportions in adaptive testing scenarios.

### 3.1 Item pool and student generation

Drawing on relevant MIRT literature (Adams et al., 1997; Hartig and Höhler, 2008), when the number of dimensions is three, it can fully examine the model's multidimensional information; therefore, the simulation study fixed the number of dimensions at three. Both studies generated student responses with an MIRT model, particularly, the multidimensional Rasch model. The multidimensional Rasch model formula is as follows (Equation 7):


$$\begin{array}{l} P\left(Y_{ij}=1|\theta_{i},\beta_{j},q_{j}\right)=\frac{exp\left(\overset{3}{\underset{m=1}{\sum }}q_{jm}\theta_{im}-\beta_{j}\right)}{1+exp\left(\overset{3}{\underset{m=1}{\sum }}q_{jm}\theta_{im}-\beta_{j}\right)} \end{array}$$ (7)

where θ_*im*_ is the m-th dimension ability for student i (*m* = 1, 2, *or* 3), and β_*j*_ is the difficulty parameter for item. If item j assesses the m-th dimension, *q*_*jm*_ = 1; otherwise, *q*_*jm*_ = 0. Under each scenario, all students' three-dimensional ability parameter vectors θ_*i*_ = (θ_*i*1_, θ_*i*2_, θ_*i*3_) were independently generated from a multivariate normal distribution *N*(μ, Σ), where the mean vector μ = [0, 0, 0]^*T*^, and the covariance matrix Σ structure was determined by the set inter-dimensional correlation, denoted as ρ. For both studies, responses were generated online based on the examinee's true latent trait vector, the selected item's Q-vector entry, and the item's true difficulty parameter.

On this basis, referencing Chen et al. (2017) and Park et al. (2019), both studies used the same two dimensional structures (between-item and within-item) but differed in pool size. In Study 1, each pool contained 90 items. The between-item pool had 30 items per dimension. The within-item pool had 9 single-dimension items (3 per dimension) and 81 two-dimension items (27 per pair). In Study 2, each pool contained 180 items. The between-item pool had 60 items per dimension. The within-item pool had six equally represented types (30 items each), three single-dimension and three two-dimension pairs. The Q-matrix structure for both pools is illustrated in Table 1.

**Table 1:** Examples of six items from two item pools with different dimensional structures.

Item	Between-item			Within-item		
	*θ1*	*θ_2_*	*θ_3_*	*θ1*	*θ_2_*	*θ_3_*
1	1	0	0	1	0	0
2	1	0	0	0	1	0
3	0	1	0	0	0	1
4	0	1	0	1	1	0
5	0	0	1	1	0	1
6	0	0	1	0	1	1

In both studies, identical item-difficulty distributions were used across the between-item and within-item pool structures; the item difficulty parameters were generated once from a standard normal distribution *N*(0, 1) at the outset of the simulation and held constant across all replications and conditions. The latent ability vectors, however, were independently regenerated in each Monte Carlo replication. In Study 1, a new sample of N examinees was drawn from a multivariate normal distribution on every replication using a replication-specific random seed; in Study 2, a fresh pool of 600 latent trait vectors were generated for each replication. Consequently, the item parameters remained fixed while the ability parameters varied independently across replications.

### 3.2 Testing procedures

Study 1 and Study 2 were designed to represent two distinct small-sample pretest scenarios. In Study 1, a linear fixed-length test was administered in which all *N* simulated examinees completed the full set of 90 items. Each examinee received an independently randomized item sequence, and examinees were processed in a person-first order (i.e., examinee *i* completed all 90 items before examinee *i*+1 began). Within the MELO procedure, provisional ability and item difficulty estimates were updated sequentially after each response. The temporal sequence and indexing scheme are shown in Algorithm 1.

In Algorithm 1, *t* indexes the global sequence of responses across all (*i, j*) pairs, incrementing from 1 to *NJ*. At each step, the provisional estimate of examinee *i*'s ability vector, denoted ${\hat{\theta }}_{i}^{\left(t\right)}$, and the provisional estimate of item *j*'s difficulty, denoted ${\hat{b}}_{j}^{\left(t\right)}$, are updated based on the observed response *X*_*ij*_ and the estimates carried forward from step *t*−1.

Study 2 represented a computer-based adaptive pretest scenario administered from an item pool of 180 items. Each examinee completed *L* items, with *N* × *L* held constant at 36,000 across conditions. The first *n*_*random*_ items for each examinee were selected randomly from the pool of unadministered items; the remaining *n*_*adaptive*_ = *L*−*n*_*random*_ items were selected via a two-stage strategy. In the first stage, candidate items were filtered to minimize the variance of dimension-specific exposure counts (i.e., a two-dimensional item contributed 0.5 to each of its loading dimensions, whereas a one-dimensional item contributed 1.0), thereby balancing measurement coverage across dimensions. In the second stage, among the balanced candidates, the item with the predicted response probability closest to 0.50 was selected (ties broken at random). No traditional item exposure control procedure (e.g., maximum exposure rate or cooling mechanism) was implemented; however, items already administered to an examinee were excluded from selection, and the dimensional balance filter indirectly promoted more uniform item usage. The administration followed a position-first processing order, meaning all examinees' responses at position *k* were processed before advancing to position *k*+1. Algorithm 2 details the corresponding indexing and update sequence.

Algorithm 1 Study 1—Person-first linear administration. 
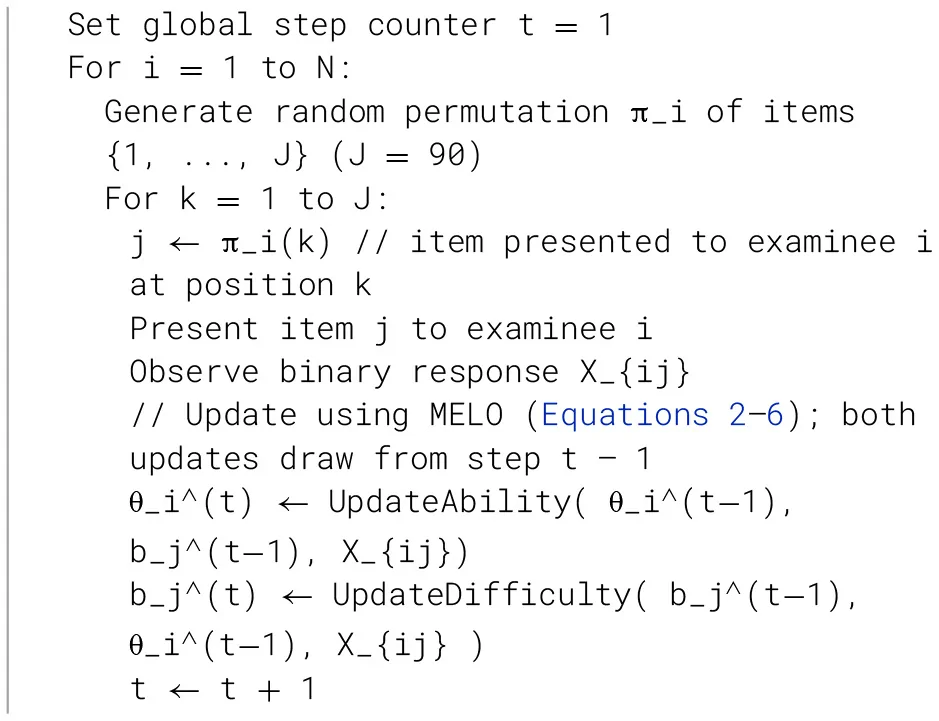

Algorithm 2 Study 2—Position-first adaptive administration 
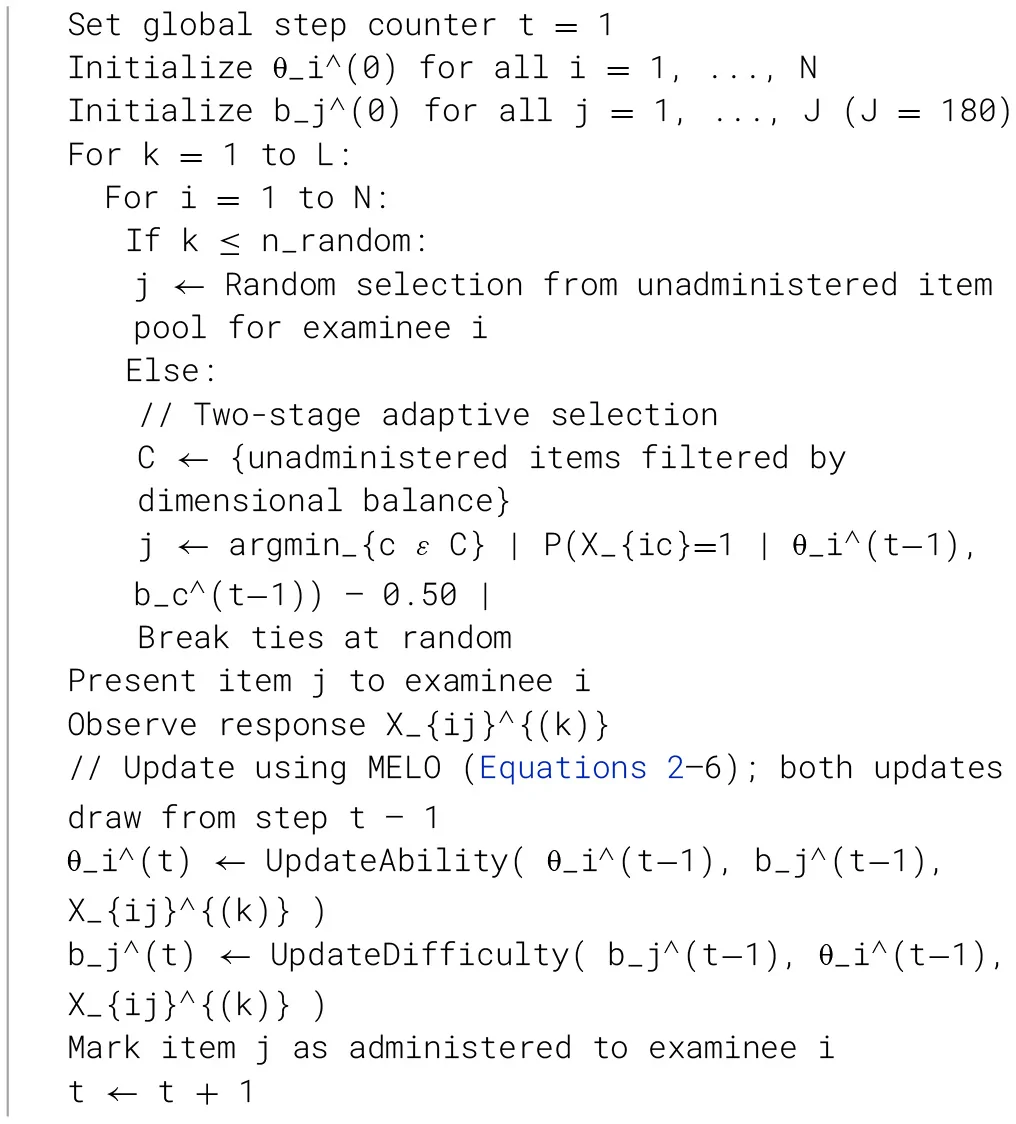

In Algorithm 2, *t* again indexes the global response sequence, but because the outer loop iterates over positions *k* and the inner loop iterates over examinees i, the temporal order is position-first: all *N* responses at position *k* are processed before any examinee advances to position *k*+1. The superscript *k* on $X_{ij}^{\left(k\right)}$ denotes the within-examinee position, whereas the superscript (*t*) on ${\hat{\theta }}_{i}^{\left(t\right)}$ and ${\hat{b}}_{j}^{\left(t\right)}$ denotes the global update step. In both algorithms, the functions UpdateAbility and UpdateDifficulty correspond to the MELO update equations described in the previous section.

### 3.3 Item calibration and ability estimation

MIRT calibration and scoring were performed using the mirt package (Chalmers, 2012) in R. A confirmatory compensatory three-dimensional Rasch model was specified according to the generating Q-matrix, with each dimension indicated by its corresponding items and the latent covariance structure freely estimated across all three dimensions; all item discriminations were constrained to 1.0 by specifying itemtype = “Rasch”. The model was fit via the Metropolis-Hastings Robbins-Monro (MHRM) algorithm with a maximum of 2,000 cycles, and convergence was determined by the optimizer's internal criteria. Because the data-generating model (using the same Q-matrix, item difficulties, and latent trait distributions) and the fitted MIRT model shared an identical parameterization, item difficulties were extracted directly. Person abilities were estimated via expected a posteriori (EAP) scoring with quasi-Monte Carlo integration, neither requiring *post-hoc* scale transformation before comparison with the generating parameters. Replications that failed to produce estimates were retained in the raw output with missing metric values, but were excluded from the computation of RMSE and bias; the convergence rate was reported as a diagnostic indicator only.

In both studies, item difficulty and examinee ability parameters were estimated concurrently using the MELO method described in the Present Study section. All item difficulty parameters and three-dimensional ability vectors were initialized at 0. After each examinee responded to an item, the corresponding item difficulty parameter and the examinee's ability vector were simultaneously updated according to the updating rules defined in Equations 3–6. An examinee's ability estimate was finalized upon completion of that examinee's test, and all item difficulty estimates were finalized after all examinees had completed the test.

### 3.4 MELO hyperparameter search and validation

The five hyperparameters (*D*, *c*_1_, *c*_2_, *d*_1_, *d*_2_) were obtained through structure-specific random searches. The random search treated the two structures completely independently, yielding two distinct parameter sets. Each search evaluated 500 candidate configurations drawn from the space defined in Table 2.

**Table 2:** Search space of MELO hyperparameters.

Parameter	Lower bound	Upper bound	Distribution
*D*	0.00	1.00	Uniform
*c_1_*	0.10	1.50	Uniform
*c_2_*	0.01	0.50	Uniform
*d_1_*	0.10	1.50	Uniform
*d_2_*	0.01	0.50	Uniform

Each candidate configuration was evaluated on a training grid of five datasets (ρ = 0, 0.3, 0.5, 0.7, 0.9; *N* = 200; five replications per cell) for its respective structure. For every cell we computed the item RMSE and ability RMSE. The primary optimization objective was a combined score defined as


$$\begin{array}{c} combined\;score=0.7\times {RMSE}_{b}+0.3\times {RMSE}_{\theta } \end{array}$$ (8)

where ${RMSE}_{b}$ and ${RMSE¯}_{\theta }$ denote the RMSE values for item difficulty and ability parameters, respectively, each averaged across the five ρ cells. This objective defined in Equation 8 reflects our prioritization of item calibration accuracy while still penalizing poor ability estimation. We first retained only candidates whose combined score fell within 1% of the best value achieved among all 500 candidates. From this eligible set, we ranked the candidates hierarchically by (a) worst-cell item RMSE, (b) combined score, (c) mean item RMSE, and (d) candidate ID, in that order, and selected the top-ranked candidate. The winning candidate for each structure was then subjected to an independent validation on fresh out-of-sample conditions (ρ = 0, 0.3, 0.5, 0.7, 0.9 crossed with *N* = 150, 250) with 50 replications each. Only after this out-of-sample validation did we lock the parameters for the main study.

A comprehensive sensitivity analysis was then conducted to ensure robustness. Specifically, we varied *D* from 0 to 1 in steps of 0.05 (21 values), crossed with 2 structures and 5 ρ values (0, 0.3, 0.5, 0.7, 0.9), with 50 replications per cell. Four decision rules were compared: no cross-dimensional updating (*D* = 0), the single value that minimizes average item RMSE across all conditions (*D* = *D*_*global*_), the values selected by structure-specific random search (*D* = *D*_*structure*−*specific*_), and the oracle rule setting the weight equal to the true data-generating correlation (*D* = ρ).

### 3.5 Simulation study design

Study 1 constructed a multi-factor experimental design integrating three key data generation conditions: two item pool structures (between-item vs. within-item multidimensionality), three inter-dimensional correlation levels (ρ = 0, 0.5, 0.8), and three sample sizes (*N* = 150, 200, 250). Study 1 constructed 18 experimental scenarios through 2 (dimensional structure) × 3 (inter-dimensional correlation) × 3 (sample size), generating 18 datasets. Based on these 18 datasets, both MELO and MIRT methods were applied for item calibration. During the item calibration phase, all students completed an identical fixed test containing 90 items, but the presentation order of items to each student was randomized (Ban et al., 2001). In Study 1, the multidimensional Rasch model served as the baseline method to evaluate the calibration effectiveness of the MELO method.

Study 2 aimed to examine the item calibration performance of the MELO method in adaptive testing scenarios. This study fixed the inter-dimensional correlation at ρ = 0.5 and controlled the average exposure frequency of each item at about 200, corresponding to the *N* = 200 condition in Study 1. To compare the estimation effects of different test combinations while controlling for total response frequency (i.e., total calibration cost), this study set four different combinations of sample size (*N*) and individual test length (*L*): *N* = 300/L = 120, *N* =4 00/L = 90, *N* =5 00/L = 72, *N* = 600/L = 60, keeping the total response frequency constant (all combinations satisfy N × L = 36,000, ensuring an average item exposure of 200). To examine the effectiveness of the MELO method under different adaptive selection proportions, we set five different adaptive selection proportions (0/3, 1/3, 1/2, 2/3, and 3/3), corresponding to gradual transitions from fully random to fully adaptive.

Each experimental condition was replicated 500 times, with final metrics averaged to ensure result robustness (Chen and Wang, 2016).

### 3.6 Simulation study evaluation criteria

For each testing scenario, this study evaluated item calibration and ability estimation accuracy using the root mean square error (RMSE; Equations 9 and 11) and bias (Equations 10 and 12). RMSE reflects the overall deviation between estimated and true values, with smaller values indicating higher accuracy; bias examines systematic estimation error and has an ideal value of 0, with positive values indicating systematic overestimation and negative values indicating underestimation. For item parameters, the RMSE and bias within each replication r are computed as


$$\begin{array}{c} RMSE_{b}^{\left(r\right)}=\sqrt{\frac{1}{J}\overset{J}{\underset{j=1}{\sum }}{\left[{\hat{b}}_{j}^{\left(r\right)}-b_{j}\right]}^{2}} \end{array}$$ (9)

$$\begin{array}{c} Bias_{b}^{\left(r\right)}=\frac{1}{J}\overset{J}{\underset{j=1}{\sum }}\left[{\hat{b}}_{j}^{\left(r\right)}-b_{j}\right] \end{array}$$ (10)

where *J* is the number of items being calibrated, ${\hat{b}}_{j}^{\left(r\right)}$ is the estimated difficulty of item j in replication r, and *b*_*j*_ is its true value. For ability parameters, the overall RMSE and bias are computed over the full N × M ability matrix,


$$\begin{array}{c} RMSE_{\theta }^{\left(r\right)}=\sqrt{\frac{1}{NM}\overset{N}{\underset{i=1}{\sum }}\overset{M}{\underset{m=1}{\sum }}{\left[{\hat{\theta }}_{im}^{\left(r\right)}-\theta_{im}\right]}^{2}} \end{array}$$ (11)

$$\begin{array}{c} Bias_{\theta }^{\left(r\right)}=\frac{1}{NM}\overset{N}{\underset{i=1}{\sum }}\overset{M}{\underset{m=1}{\sum }}\left[{\hat{\theta }}_{im}^{\left(r\right)}-\theta_{im}\right] \end{array}$$ (12)

with N examinees and M = 3 dimensions; dimension-specific RMSEs are defined analogously by restricting the sum to a single dimension m.

To characterize how estimation accuracy varies across the ability and difficulty continua, conditional (grouped) RMSE (Equations 13 and 15) and bias (Equations 14 and 16) were additionally computed. Within each replication, students were divided separately on each dimension m into six groups according to their true abilities, using the fixed cut points −2, −1, 0, 1, and 2 [i.e., (–∞, −2], (−2, −1], (−1, 0], (0, 1], (1, 2], and (2, +∞)]. The conditional RMSE and bias for dimension m and ability group g are


$$\begin{array}{c} RMSE_{\theta }^{\left(r,m,g\right)}=\sqrt{\frac{1}{\left|G_{gm}^{\left(r\right)}\right|}\underset{i\in G_{gm}^{\left(r\right)}}{\sum }{\left[{\hat{\theta }}_{im}^{\left(r\right)}-\theta_{im}\right]}^{2}} \end{array}$$ (13)

$$\begin{array}{c} Bias_{\theta }^{\left(r,m,g\right)}=\frac{1}{\left|G_{gm}^{\left(r\right)}\right|}\underset{i\in G_{gm}^{\left(r\right)}}{\sum }\left[{\hat{\theta }}_{im}^{\left(r\right)}-\theta_{im}\right] \end{array}$$ (14)

where $G_{gm}^{\left(r\right)}$ denotes the set of students whose true ability on dimension m falls into group *g* in replication r. Likewise, items were divided into six groups according to their true difficulties using the same fixed cut points, and the conditional RMSE and bias for difficulty group *g* are


$$\begin{array}{c} RMSE_{b}^{\left(r,g\right)}=\sqrt{\frac{1}{\left|H_{g}\right|}\underset{j\in H_{g}^{\left(r\right)}}{\sum }{\left[{\hat{b}}_{j}^{\left(r\right)}-b_{j}\right]}^{2}} \end{array}$$ (15)

$$\begin{array}{c} Bias_{b}^{\left(r,g\right)}=\frac{1}{\left|H_{g}\right|}\underset{j\in H_{g}^{\left(r\right)}}{\sum }\left[{\hat{b}}_{j}^{\left(r\right)}-b_{j}\right] \end{array}$$ (16)

where *H*_*g*_ denotes the set of items whose true difficulties fall into group g; because the true item parameters were held constant across replications, *H*_*g*_ was identical in every replication. If an ability group happened to contain no students in a given replication, that replication did not contribute to the corresponding conditional metric.

All RMSE and bias values, both overall and conditional, are means of these replication-level quantities across the 500 replications of each condition. The Monte Carlo standard error (MCSE) of each reported metric was calculated as the standard deviation across replications divided by the square root of 500. For any replication-level quantity *Q*^(*r*)^ (e.g., $RMSE_{b}^{\left(r\right)}$, $RMSE_{\theta }^{\left(r\right)}$, or their conditional counterparts), the Monte Carlo standard error of its mean $\overset{¯}{Q}=\left(1/R\right)\overset{R}{\underset{r=1}{\sum }}Q^{\left(r\right)}$ was computed as


$$\begin{array}{c} MCSE\left(\overset{¯}{Q}\right)=\sqrt{\frac{1}{R\left(R-1\right)}\overset{R}{\underset{r=1}{\sum }}{\left(Q^{\left(r\right)}-\overset{¯}{Q}\right)}^{2}} \end{array}$$ (17)

where *R* = 500 denotes the number of replications. The same formula (Equation 17) applied to both overall and conditional metrics, with the latter computed only over replications in which the relevant group contained at least one observation.

The comparison of RMSE between MELO and MIRT is paired at the replication level: MELO and MIRT are fitted to identical response matrices drawn from the same deterministic seeds, and the primary comparison uses within-replication paired differences with 95% Monte Carlo confidence intervals.

To examine whether the order in which examinees entered the system affected ability estimation accuracy in Study 1, examinees were divided into four groups—Q1, Q2, Q3, and Q4—based on their position in the processing sequence, with group sizes kept as equal as possible. Q1 comprised the earliest examinees processed, whereas Q4 comprised the latest. This grouping was determined solely by processing order and was independent of examinees' true or estimated abilities. For each group, overall and dimension-specific ability RMSEs and corresponding bias values were computed to evaluate whether estimation accuracy varied as a function of entry order.

For Study 2, item exposure was summarized by its median, coefficient of variation, and the proportion of items receiving fewer than 100 administrations.

### 3.7 Preliminary empirical illustration

To evaluate whether the online joint estimation procedure of the multidimensional Elo (MELO) algorithm could recover item difficulty parameters and latent trait estimates converging with those from a conventional multidimensional Rasch model fitted via marginal maximum likelihood, the publicly available SAT12 dataset (included in the mirt R package) was analyzed. The SAT12 is a 12th-grade science assessment distributed with the TESTFACT software manual and accessible through the R package mirt (Chalmers, 2012). It comprises 32 dichotomously scored items (*J* = 32) administered to 600 examinees (*N* = 600), with 572 complete response vectors commonly retained for analysis. Items map onto three between-dimensional content domains: Chemistry (*n* = 13), Biology (*n* = 7), and Physics (*n* = 12).

We applied MELO using the hyperparameters obtained from the random search in Study 1 and compared the resulting estimates with those from a multidimensional Rasch model fitted via mirt (EM algorithm, EAP scoring). The Pearson correlations between MELO and MIRT item-difficulty estimates and dimension-specific ability estimates were calculated.

## 4 Results

### 4.1 Hyperparameter selection and robustness evaluation

Independent random searches identified the final MELO parameter configurations for the two item structures. For the between-item multidimensional structure, the selected values were *D* = 0.2061, *c*_1_ = 1.3094, *c*_2_ = 0.0788, *d*_1_ = 1.3809, and *d*_2_ = 0.1561; for the within-item multidimensional structure, the values were *D* = 0.4804, *c*_1_ = 0.5150, *c*_2_ = 0.0251, *d*_1_ = 1.2020, and *d*_2_ = 0.1826. These configurations were used in both simulation studies and the real-data demonstration.

Figure 1 displays the sensitivity of raw item RMSE (left panel) and raw ability RMSE (right panel) to the cross-dimensional update weight D, separately for the between-item and within-item structures and across five levels of ρ. For item calibration (left panel), the between-item structure exhibited a monotonic increase in RMSE as D increased. By contrast, the within-item structure showed minimal sensitivity to D. For ability estimation (right panel), RMSE increased with D for both structures, though the magnitude of this increase depended strongly on ρ. At D = 0, ability RMSE values ranged from approximately 0.38 to 0.43 across all conditions. For the between-item structure, ability RMSE rose sharply with D at high ρ but remained nearly flat at ρ = 0. The within-item structure followed a similar pattern, albeit with smaller increases at high ρ and minimal change at low ρ. The selected hyperparameters are consistent with these results. D = 0.2061 for the between-item structure sits near the region of optimal item RMSE; for the within-item structure, D = 0.4804 corresponds to a region where both item and ability RMSE remain well-controlled across ρ levels.

**Figure 1:**
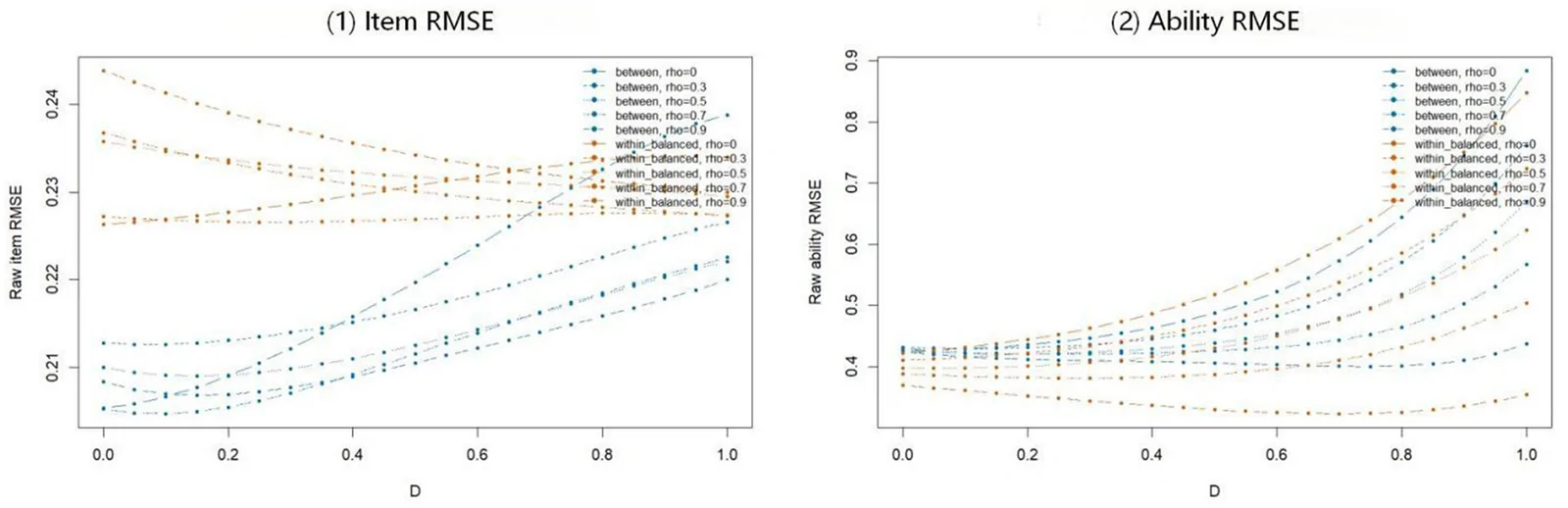
Item and ability RMSE as a function of cross-dimensional update weight (D) for between-item and within-item structures across ability correlation levels.

Table 3 reports item difficulty RMSEs under alternative cross-dimensional update weight (D) rules. For the between-item structure, item RMSE was lowest with the structure-specific D at ρ = 0.30, 0.50, and 0.70, and differences among the four rules were modest overall. For the within-item structure, the structure-specific D and the D = ρ rule performed comparably and consistently outperformed the D = 0 baseline and the global constant D = 0.15 across all ρ levels. Across both structures, the structure-specific D values provided a generally robust compromise, never yielding the highest RMSE in any condition. These results support the decision to use structure-specific D values in the main simulation studies.

**Table 3:** Item difficulty RMSE under alternative cross-dimensional update weight (D) rules by dimensional structure and ability correlation.

Dimensional structure	ρ	*D* = 0	Global *D* = 0.15	Structure-specific *D*	*D = ρ*
Between-item	0	0.2137	0.2151	0.2160	0.2137
	0.3	0.2110	0.2095	0.2094	0.2098
	0.5	0.2103	0.2082	0.2078	0.2083
	0.7	0.2066	0.2053	0.2051	0.2072
	0.9	0.2140	0.2116	0.2113	0.2163
Within-item	0	0.2327	0.2334	0.2363	0.2327
	0.3	0.2294	0.2279	0.2265	0.2270
	0.5	0.2290	0.2272	0.2253	0.2252
	0.7	0.2421	0.2396	0.2360	0.2346
	0.9	0.2458	0.2420	0.2360	0.2316

Global D = 0.15 was determined by minimizing the average item RMSE across both structures and all five ρ levels from the D-sensitivity results. Structure-specific D values were 0.2061 for the between-item structure and 0.4804 for the within-item structure. All values are means across 50 replications per condition.

### 4.2 Study 1: comparison of item calibration between MELO and MIRT

#### 4.2.1 RMSE and bias of estimated item difficulties

Table 4 summarizes the RMSE of item calibration of MELO and MIRT under different sample sizes (N), inter-dimensional correlations (ρ), and dimensional structures (between-item vs. within-item multidimensionality).

**Table 4:** Item difficulty RMSE, Monte Carlo standard error, and 95% confidence intervals for method differences by sample size, dimensional correlation, and dimensional structure.

*N*	ρ	Between-item			Within-item		
		MELO	MIRT	95% CI	MELO	MIRT	95% CI
150	0	0.2382 (0.0011)	0.2199 (0.0010)	[0.0161, 0.0206]	0.2539 (0.0012)	0.2382 (0.0014)	[0.0126, 0.0187]
	0.5	0.2359 (0.0011)	0.2174 (0.0011)	[0.0161, 0.0209]	0.2561 (0.0015)	0.2582 (0.0022)	[−0.0071, 0.0029]
	0.8	0.2342 (0.0013)	0.2280 (0.0014)	[0.0037, 0.0096]	0.2559 (0.0015)	0.2646 (0.0023)	[−0.0138,−0.0044]
200	0	0.2087 (0.0011)	0.1871 (0.0008)	[0.0195, 0.0236]	0.2298 (0.0011)	0.2052 (0.0012)	[0.0217, 0.0275]
	0.5	0.2061 (0.0011)	0.1875 (0.0009)	[0.0163, 0.0209]	0.2295 (0.0013)	0.2192 (0.0016)	[0.0063, 0.0141]
	0.8	0.2089 (0.0012)	0.1931 (0.0011)	[0.0127, 0.0183]	0.2292 (0.0014)	0.2273 (0.0020)	[−0.0022, 0.0063]
250	0	0.1900 (0.0010)	0.1683 (0.0007)	[0.0197, 0.0237]	0.2131 (0.0011)	0.1831 (0.0011)	[0.0273, 0.0327]
	0.5	0.1888 (0.0010)	0.1679 (0.0008)	[0.0187, 0.0229]	0.2124 (0.0013)	0.1961 (0.0016)	[0.0122, 0.0203]
	0.8	0.1874 (0.0011)	0.1766 (0.0011)	[0.0082, 0.0137]	0.2162 (0.0014)	0.2054 (0.0019)	[0.0065, 0.0152]

Values in parentheses are Monte Carlo standard errors (MCSE). MELO, multidimensional Elo; MIRT, marginal maximum likelihood estimation with multidimensional item response theory. All values are means across 500 replications per condition.

As shown in Table 4, under the between-item structure, MIRT produced significantly smaller item difficulty RMSEs than MELO across all conditions; all the confidence intervals for the paired differences excluded zero. Under the within-item structure, however, this advantage attenuated as the inter-dimensional correlation increased: the difference became statistically non-significant in some conditions and even reversed in favor of MELO at *N* = 150 and ρ = 0.80, 95% CI [−0.0138, −0.0044].

For both structures, a higher inter-dimensional correlation narrowed the gap between the two methods. Under the within-item structure, this narrowing was driven by a monotonic increase in the RMSE of MIRT with ρ, whereas the RMSE of MELO remained essentially flat. Under the between-item structure, MIRT RMSE was comparatively stable at ρ ≤ 0.50 but increased at ρ = 0.80.

Increasing sample size also improved calibration accuracy: as N increased from 150 to 250, the RMSE decreased under all conditions for both methods (Table 4). This increase in sample size further strengthened MIRT's relative advantage.

Caution is warranted, however, in interpreting MIRT's advantage. MIRT estimation did not always converge or yield admissible parameter estimates under high-correlation conditions (Table 5). Table 5 reports the success rate (percentage of replications yielding admissible estimates) and the convergence rate (percentage meeting formal convergence criteria) for each condition. The gap between these rates reflects replications that produced admissible estimates despite failing to meet formal convergence criteria.

**Table 5:** Convergence performance of MIRT by dimensional structure, sample size, and dimensional correlation.

*N*	ρ	Between-item		Within-item	
		Success rate (%)	Convergence rate (%)	Success rate (%)	Convergence rate (%)
150	0	100.00	100.00	100.00	99.60
	0.5	100.00	99.60	100.00	99.80
	0.8	85.20	25.60	92.20	51.20
200	0	100.00	100.00	100.00	100.00
	0.5	100.00	100.00	99.80	99.80
	0.8	88.80	36.80	98.00	71.00
250	0	100.00	100.00	100.00	100.00
	0.5	99.80	99.80	100.00	100.00
	0.8	87.20	29.00	98.80	63.20

Success rate, percentage of replications yielding admissible parameter estimates. Convergence rate, percentage of replications in which the estimation algorithm met formal convergence criteria; MIRT, multidimensional item response theory. All values are percentages rounded to two decimal places.

As shown in Table 5, estimation performance was excellent at low to moderate correlations (ρ = 0 and ρ = 0.50), with success and convergence rates approaching or reaching 100% across all conditions. Substantial deterioration emerged only at ρ = 0.80, particularly for the between-item structure, whereas the within-item structure proved more robust. Increasing the sample size yielded modest improvements under the high-correlation condition; for example, within-item convergence rose from 51.20% at *N* = 150 to 63.20% at *N* = 250.

To examine how calibration accuracy varies along the difficulty continuum, item difficulty RMSE was computed conditional on true difficulty. Figure 2 presents the results for *N* = 200; patterns at the other sample sizes were virtually identical.

**Figure 2:**
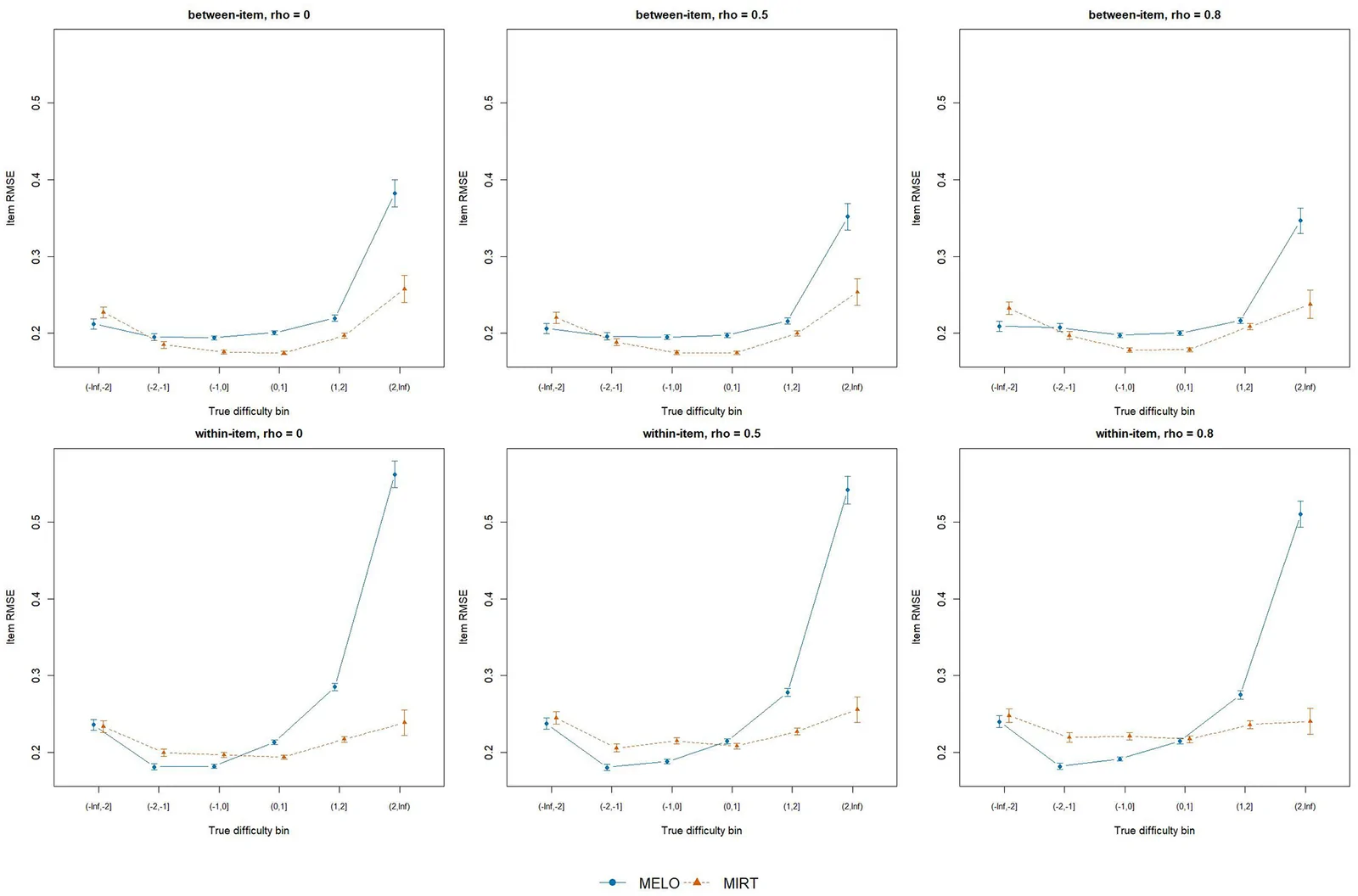
Item difficulty RMSE conditional on true difficulty, comparing MELO and MIRT by dimensional structure and dimensional correlation (*N* = 200). RMSE, root mean square error; MELO, multidimensional Elo; MIRT, multidimensional item response theory. Points and error bars represent means and 95% Monte Carlo confidence intervals across 500 replications. Rows correspond to the dimensional structure (between-item vs. within-item) and columns to the inter-dimensional correlation (ρ = 0, 0.5, 0.8). True difficulty was binned with cutpoints at −2, −1, 0, 1, and 2. Patterns at *N* = 150 and *N* = 250 were virtually identical and are not shown.

The MIRT curves were mildly U-shaped in all conditions: RMSE was slightly elevated in the two extreme bins and lowest for items of moderate difficulty. MELO exhibited a pronounced spike in the most difficult bin (b > 2). This small group of extremely difficult items accounts for much of MELO's higher overall item RMSE reported in Table 4. Excluding the b > 2 bin, the two methods differed only modestly under either dimensional structure.

Under the between-item structure, MIRT's RMSE was lower across most difficulty levels, with the sole exception of the easiest bin (b ≤ −2), where MELO was slightly more accurate. Under the within-item structure, relative performance depended on item difficulty: MELO achieved lower RMSE for easier items (b ≤ 0), the two methods were nearly tied in the intermediate (0,1] bin, and MIRT was more accurate for items with b > 1, with the gap widening sharply in the extreme bin.

Figure 3 plots the conditional bias of item difficulty estimation by true-difficulty bin at *N* = 200, separately for the between- and within-item structures and the three correlation levels; this sample size is representative, as the bias patterns were essentially identical at *N* = 150 and 250.

**Figure 3:**
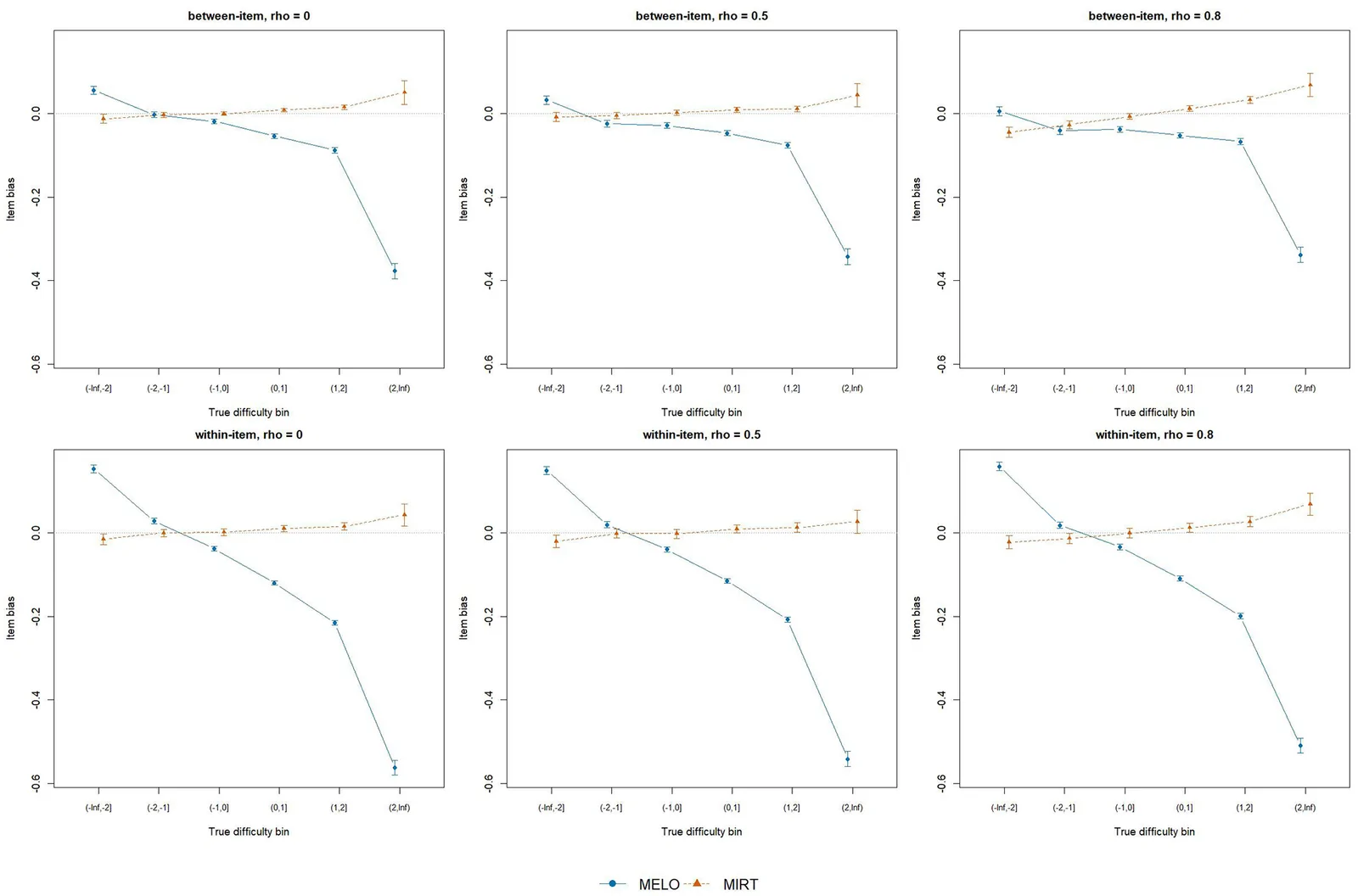
Item difficulty bias conditional on true difficulty, comparing MELO and MIRT by dimensional structure and dimensional correlation (*N* = 200). MELO, multidimensional Elo; MIRT, multidimensional item response theory. Points and error bars represent means and 95% Monte Carlo confidence intervals across 500 replications. Rows correspond to the dimensional structure (between-item vs. within-item) and columns to the inter-dimensional correlation (ρ = 0, 0.5, 0.8). True difficulty was binned with cutpoints at −2, −1, 0, 1, and 2. Patterns at *N* = 150 and *N* = 250 were virtually identical and are not shown.

The two methods exhibited markedly different patterns in Figure 3. MIRT's conditional bias remained near zero in every bin and condition, showing only a slight tendency toward underestimation at the easy end and overestimation at the hard end. MELO, in contrast, displayed an inward (regression-to-the-mean) bias pattern: it slightly overestimated the difficulty of the easiest items, was essentially unbiased for items of moderate difficulty, and increasingly underestimated difficulty as items became harder. This underestimation was already visible in the (1, 2] bin and was most pronounced in the (2, ∞) bin. The inward pattern was markedly steeper under the within-item structure.

These conditional patterns also qualify how the RMSE results reported in Table 4 should be interpreted. For MIRT, bias was negligible in every bin, so its RMSE essentially reflects sampling variability alone. For MELO, bias was likewise small across the easy-to-moderate range, and its RMSE in that range is therefore also primarily a measure of precision; however, at the difficult end, MELO's RMSE partly reflects this systematic underestimation rather than random error alone. In other words, except for the most difficult items under MELO, the RMSE differences between the two methods primarily reflect differences in estimation precision rather than systematic error.

#### 4.2.2 RMSE and bias of estimated abilities

Table 6 summarizes the RMSE of ability estimation of MELO and MIRT. For between-item structures, MELO consistently yielded slightly higher RMSE than MIRT across all sample sizes and correlation conditions, with confidence intervals above zero. In contrast, for within-item structures, a pronounced interaction between estimation method and dimensional correlation emerged. At low to moderate correlations (ρ = 0.0 and ρ = 0.5), MELO produced substantially larger RMSE than MIRT. However, this pattern reversed at high correlation (ρ = 0.8). These patterns were consistent across sample sizes (*N* = 150, 200, 250).

**Table 6:** Ability RMSE, Monte Carlo standard error, and 95% confidence intervals for method differences by sample size, dimensional correlation, and dimensional structure.

*N*	ρ	Between-item			Within-item		
		MELO	MIRT	95% CI	MELO	MIRT	95% CI
150	0	0.4391 (0.0008)	0.4119 (0.0007)	[0.0258, 0.0285]	0.5123 (0.0009)	0.3825 (0.0008)	[0.1283, 0.1312]
	0.5	0.4261 (0.0009)	0.3987 (0.0007)	[0.0258, 0.0291]	0.4306 (0.0007)	0.3761 (0.0008)	[0.0531, 0.0560]
	0.8	0.4191 (0.0009)	0.4036 (0.0015)	[0.0132, 0.0191]	0.3610 (0.0007)	0.3997 (0.0050)	[−0.0484,−0.0288]
200	0	0.4364 (0.0007)	0.4090 (0.0006)	[0.0263, 0.0286]	0.5107 (0.0008)	0.3805 (0.0006)	[0.1289, 0.1316]
	0.5	0.4224 (0.0008)	0.3966 (0.0006)	[0.0245, 0.0271]	0.4288 (0.0007)	0.3717 (0.0006)	[0.0561, 0.0581]
	0.8	0.4169 (0.0009)	0.3923 (0.0014)	[0.0217, 0.0278]	0.3606 (0.0006)	0.3709 (0.0037)	[−0.0177,−0.0029]
250	0	0.4338 (0.0007)	0.4077 (0.0005)	[0.0250, 0.0271]	0.5094 (0.0007)	0.3792 (0.0006)	[0.1291, 0.1314]
	0.5	0.4213 (0.0007)	0.3950 (0.0005)	[0.0250, 0.0274]	0.4287 (0.0006)	0.3707 (0.0006)	[0.0570, 0.0589]
	0.8	0.4141 (0.0007)	0.3957 (0.0014)	[0.0156, 0.0214]	0.3603 (0.0006)	0.3797 (0.0038)	[−0.0268,−0.0119]

Values in parentheses are Monte Carlo standard errors (MCSE). MELO, multidimensional Elo; MIRT, marginal maximum likelihood estimation with multidimensional item response theory. All values are means across 500 replications per condition.

Figure 4 displays ability RMSE conditional on true theta for *N* = 200, disaggregating the overall accuracy patterns reported in Table 6 across the ability continuum. Under between-item structures, both methods produced U-shaped RMSE profiles with elevated errors at the ability extremes and minima near the distribution center. However, under within-item structures, the conditional profiles revealed substantial heterogeneity. At low and moderate dimensional correlations (ρ = 0.0 and ρ = 0.5), MELO exhibited severely inflated RMSE at both tails, while MIRT maintained substantially lower errors in these extreme regions. At high correlation (ρ = 0.8), this tail-specific inflation in estimation error for MELO was largely attenuated.

**Figure 4:**
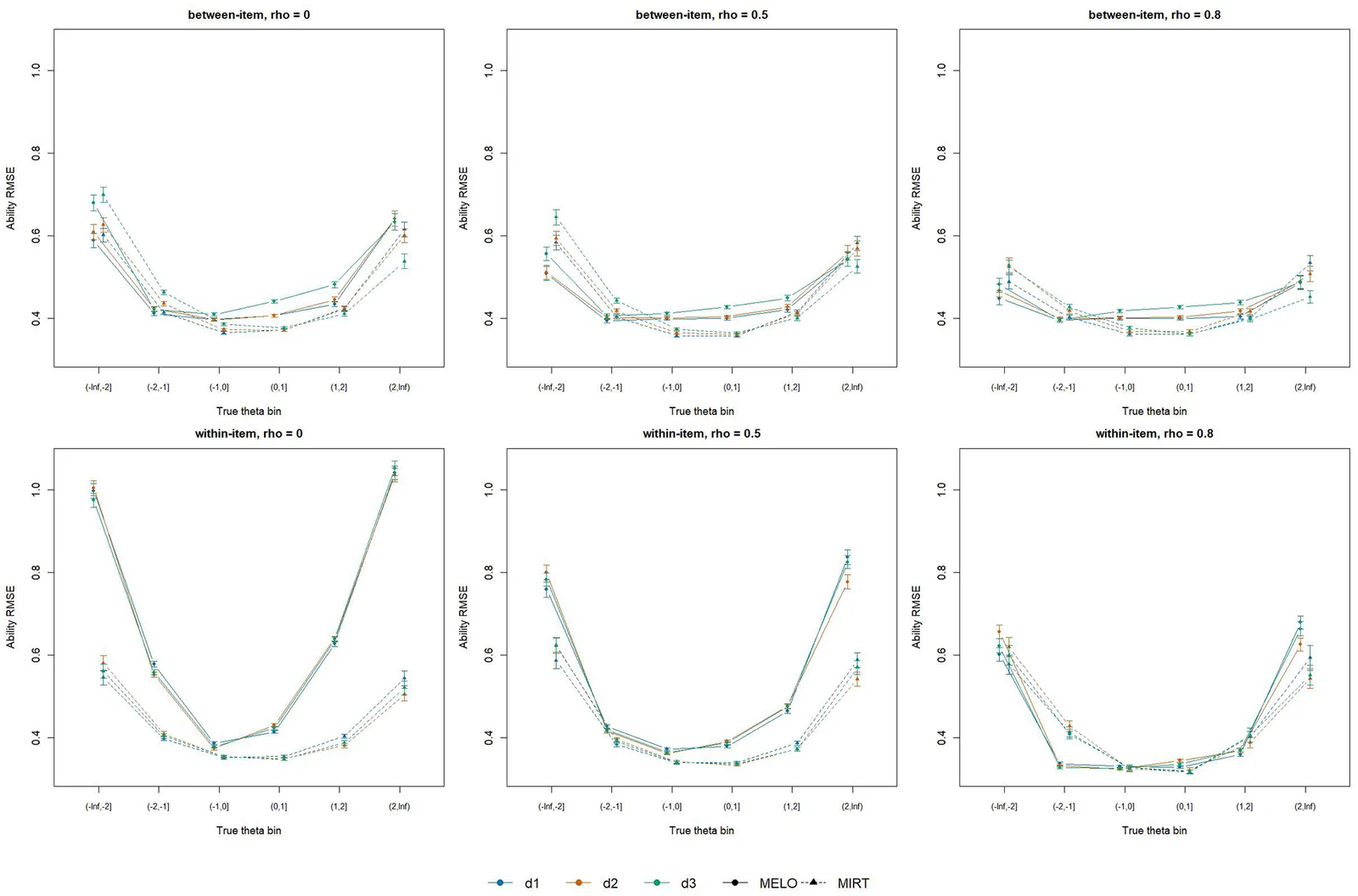
Ability RMSE conditional on true ability, comparing MELO and MIRT by dimensional structure and dimensional correlation (*N* = 200). RMSE, root mean square error; MELO, multidimensional Elo; MIRT, multidimensional item response theory. Points and error bars represent means and 95% Monte Carlo confidence intervals across 500 replications. Rows correspond to the dimensional structure (between-item vs. within-item) and columns to the inter-dimensional correlation (ρ = 0, 0.5, 0.8). True ability was binned with cutpoints at −2, −1, 0, 1, and 2. Patterns at *N* = 150 and *N* = 250 were virtually identical and are not shown.

The effect of examinee entry order on overall ability estimation accuracy is summarized in Appendix A. Table A1 in Appendix A reports the overall ability RMSE for each quartile of the entry sequence (Q1–Q4). Results suggest that later-processed examinees generally yielded slightly more accurate ability estimates.

Figure 5 plots the conditional bias of ability estimates by true-θ bin at *N* = 200, separately for the three dimensions (d1–d3), the two dimensional structures, and the three correlation levels; patterns at *N* = 150 and *N* = 250 were essentially the same. Both methods exhibited the inward (regression-to-the-mean) bias pattern: ability was overestimated for low-ability examinees, underestimated for high-ability examinees. The three dimensions were virtually indistinguishable within each panel.

**Figure 5:**
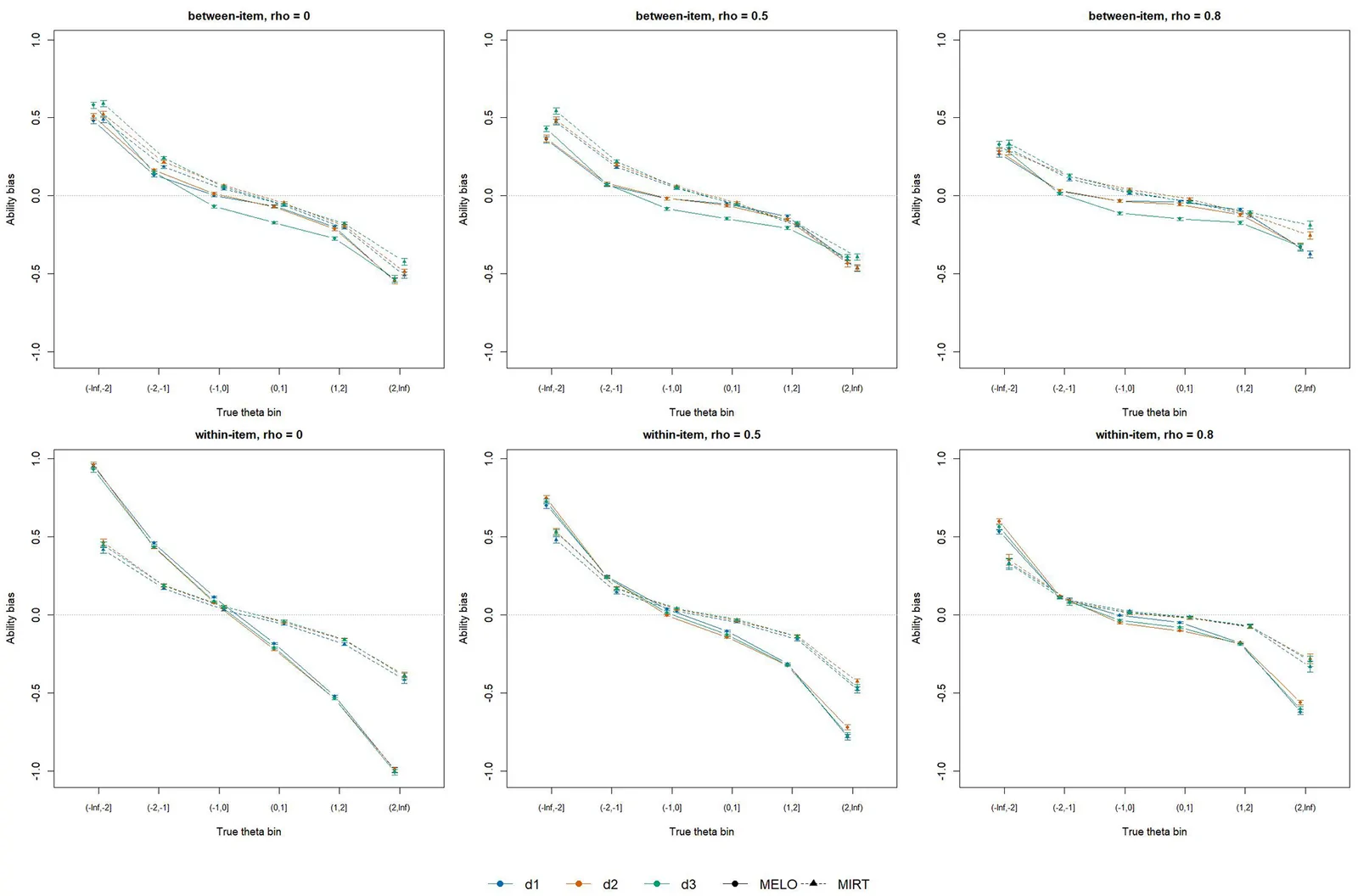
Ability bias conditional on true ability, comparing MELO and MIRT by dimensional structure and dimensional correlation (*N* = 200). MELO, multidimensional Elo; MIRT, multidimensional item response theory. Points and error bars represent means and 95% Monte Carlo confidence intervals across 500 replications. Rows correspond to the dimensional structure (between-item vs. within-item) and columns to the inter-dimensional correlation (ρ = 0, 0.5, 0.8). True ability was binned with cutpoints at −2, −1, 0, 1, and 2. Patterns at *N* = 150 and *N* = 250 were virtually identical and are not shown.

Under the between-item structure (see the upper panel of Figure 5), MELO and MIRT were nearly identical and the slope flattened as the correlation increased (at ρ = 0.8). Under the within-item structure (see the lower panel of Figure 5), in contrast, MELO's inward bias was markedly steeper than MIRT's, especially when the dimensions were uncorrelated. This gap attenuated as the correlation increased but did not disappear. For examinees at extreme θ, bias of this magnitude constitutes a non-trivial component of estimation error, particularly for MELO under the within-item structure, so RMSE differences at the extremes partly reflect differential shrinkage rather than precision alone.

### 4.3 Study 2: item calibration performance of MELO in adaptive testing scenarios

#### 4.3.1 RMSE and bias of estimated item difficulties

Table 7 presents the item difficulty RMSE and MCSE across sample sizes, test lengths, adaptive selection proportions, and dimensional structures. For the between-item multidimensional structure, when the total number of item responses (*N*×*L*) was held constant, different combinations of *N* and *L* under fully random item selection yielded negligible differences in item calibration accuracy, with RMSE values remaining stable at approximately 0.188 to 0.189. However, upon the introduction of adaptive item selection, RMSE demonstrated a monotonic increase as sample size increased from *N* = 300/*L*= 120 to *N* = 600/*L*= 60. Within each *N* and *L* combination, higher proportions of adaptive item selection were associated with elevated RMSE values, with fully adaptive selection producing the largest RMSE (ranging from 0.4216 to 0.5620).

**Table 7:** Item difficulty RMSE and Monte Carlo standard error of MELO by sample size–test length combination, adaptive selection proportions, and dimensional structure.

Dimensional structure	Adaptive selection proportion	*N* = 300/L = 120	*N* = 400/L = 90	*N* = 500/L = 72	*N* = 600/L = 60
Between-item	0/3 (random)	0.1891 (0.0005)	0.1883 (0.0005)	0.1881 (0.0005)	0.1883 (0.0005)
	1/3	0.1939 (0.0005)	0.1949 (0.0005)	0.1963 (0.0005)	0.1977 (0.0005)
	1/2	0.2037 (0.0006)	0.2097 (0.0006)	0.2134 (0.0005)	0.2167 (0.0005)
	2/3	0.2234 (0.0006)	0.2404 (0.0006)	0.2499 (0.0007)	0.2554 (0.0007)
	3/3 (full)	0.4216 (0.0010)	0.5003 (0.0010)	0.5416 (0.0012)	0.5620 (0.0013)
Within-item	0/3 (random)	0.1972 (0.0005)	0.1998 (0.0006)	0.2026 (0.0005)	0.2062 (0.0005)
	1/3	0.1904 (0.0005)	0.1905 (0.0005)	0.1907 (0.0005)	0.1922 (0.0005)
	1/2	0.1876 (0.0005)	0.1875 (0.0005)	0.1887 (0.0005)	0.1891 (0.0005)
	2/3	0.1884 (0.0005)	0.1857 (0.0006)	0.1863 (0.0005)	0.1859 (0.0005)
	3/3 (full)	0.2303 (0.0009)	0.2214 (0.0008)	0.2102 (0.0008)	0.2022 (0.0007)

RMSE, Root Mean Squared Error; MCSE, Monte Carlo standard error; values in parentheses are MCSEs; N, sample size; L, test length. The inter-dimensional correlation was fixed at ρ = 0.50 across all conditions; 0/3 (random), fully random selection; 1/3, 1/2, 2/3, adaptive selection proportions; 3/3 (full), fully adaptive selection. All values are means across 500 replications.

In contrast, for the within-item multidimensional structure, RMSE did not exhibit a consistent pattern of change across the *N* and *L* combinations. Regarding the effect of adaptive selection proportion, RMSE decreased initially as adaptive selection was introduced, reaching its lowest values at the 1/2 or 2/3 adaptive proportion, and subsequently increased at higher adaptive proportions, with fully adaptive selection again yielding elevated RMSE values relative to the intermediate adaptive conditions.

Figure 6 complements Table 7 by illustrating the conditional calibration error across the difficulty continuum. For the between-item structure (upper panel), a pronounced U-shaped pattern emerged for the fully adaptive condition (prop = 1), with RMSE markedly escalating at both extremes of the difficulty distribution. This effect was substantially attenuated for all partial-adaptive proportions (prop = 1/3, 1/2, 2/3) and the fully random condition (prop = 0), which exhibited relatively flat and low RMSE curves. In contrast, for the within-item structure (lower panel), the differentiation among adaptive selection proportions was considerably muted. All conditions displayed a modest U-shaped pattern, with slightly elevated RMSE at the extreme difficulty bins and lower error near the center.

**Figure 6:**
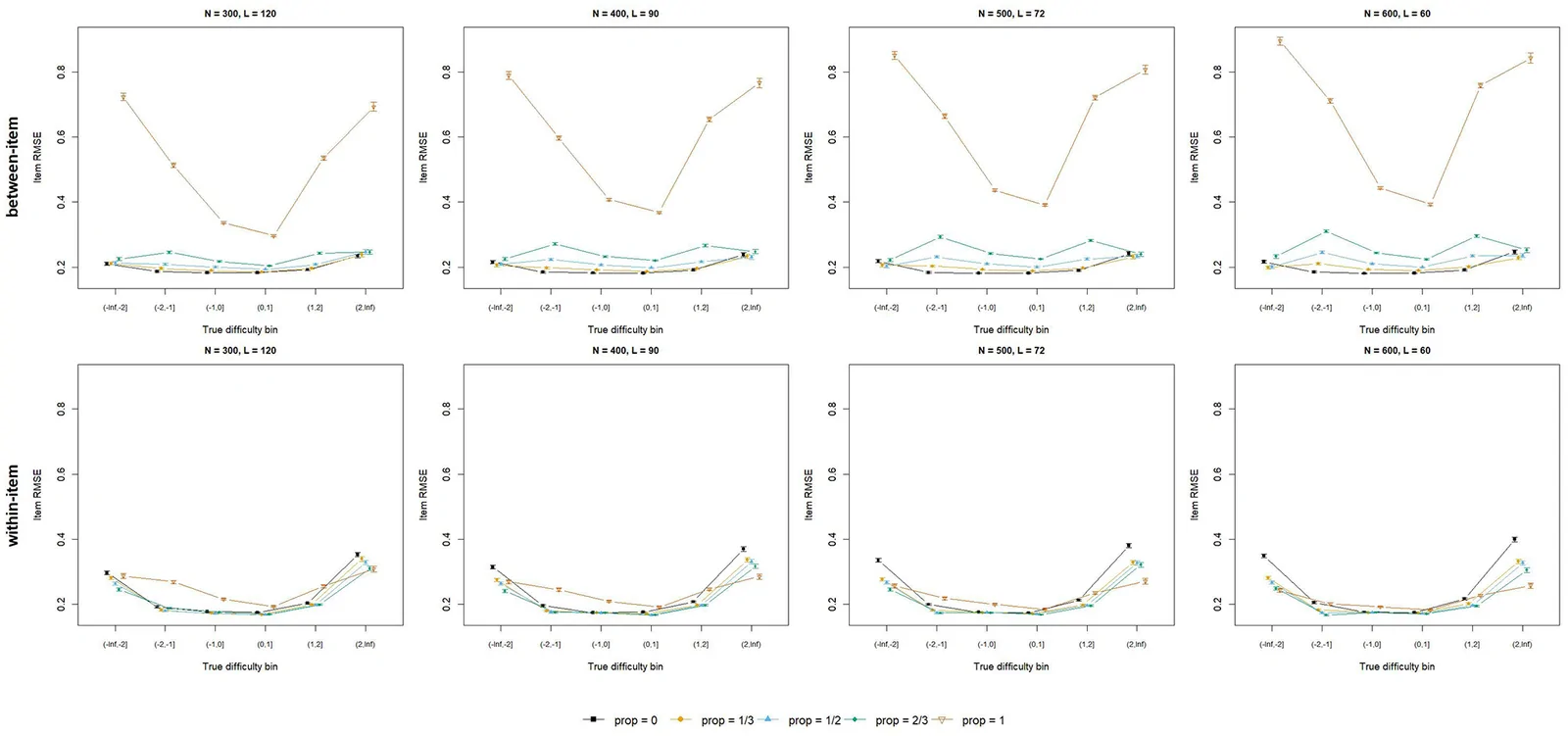
Item difficulty RMSE of MELO conditional on true difficulty, comparing different adaptive selection proportions by sample size–test length combination and dimensional structure (ρ = 0.5). RMSE, root mean square error; MELO, multidimensional Elo. Points and error bars represent means and 95% Monte Carlo confidence intervals across 500 replications. Rows correspond to the dimensional structure (between-item vs. within-item) and columns to the sample size–test length combination (*N* = 300/L = 120, *N* = 400/L = 90, *N* = 500/L = 72, and *N* = 600/L = 60). True difficulty was binned with cutpoints at −2, −1, 0, 1, and 2.

To facilitate interpretation of the RMSE findings, Figure 7 presents the conditional item difficulty bias of MELO. Under fully random item selection (prop = 0), both dimensional structures exhibited a regression-to-the-mean pattern, wherein easy items (left-tail bins) were positively biased and difficult items (right-tail bins) were negatively biased. This inward bias was more pronounced in the within-item structure than in the between-item structure. Conversely, under fully adaptive selection (prop = 1), the between-item structure displayed a marked outward bias pattern, where easy items were substantially underestimated (negative bias) and difficult items were overestimated (positive bias). The magnitude of the bias increased as sample size increased from *N* = 300 to *N* = 600. The within-item structure, by contrast, showed only a modest outward bias under full adaptation.

**Figure 7:**
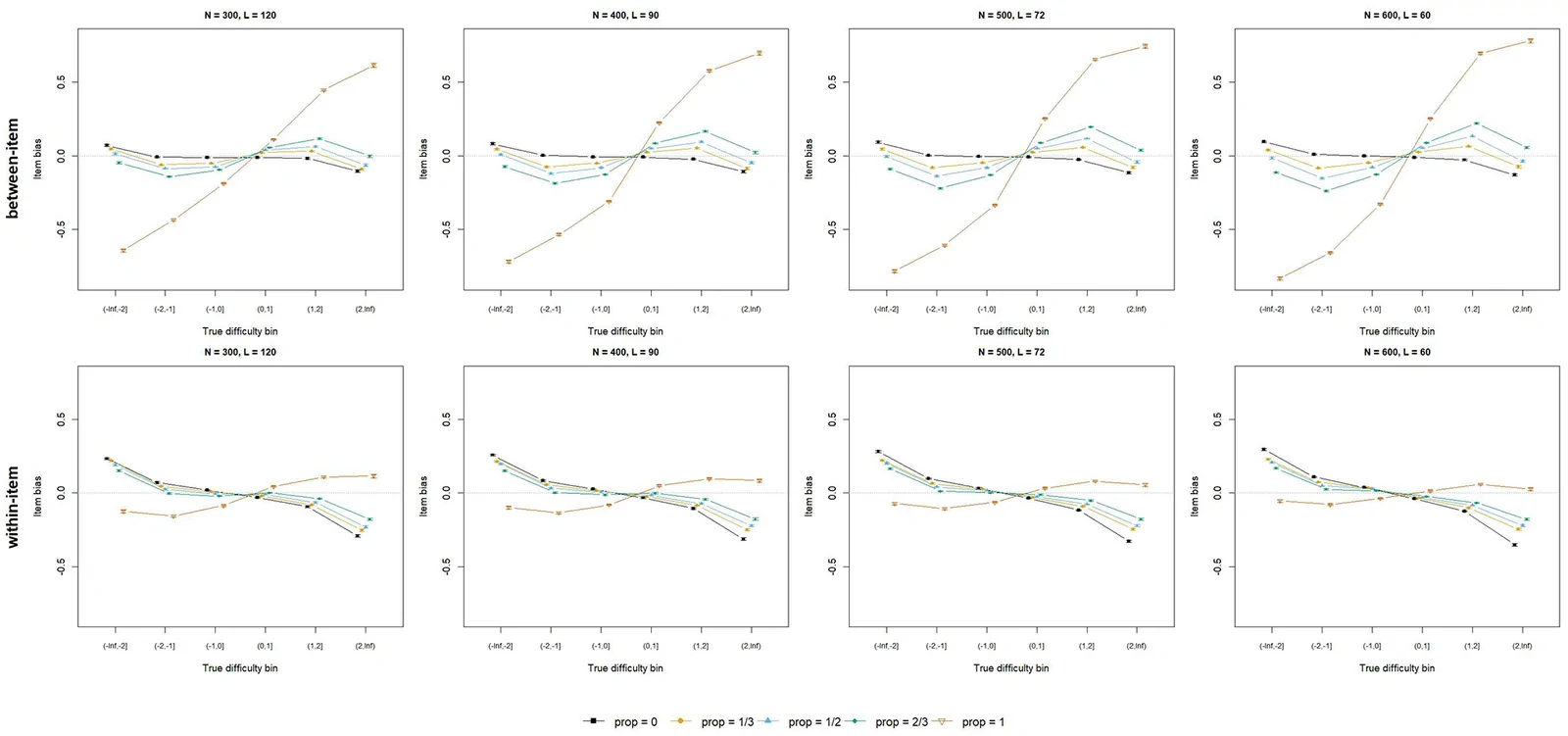
Item difficulty bias of MELO conditional on true difficulty, comparing different adaptive selection proportions by sample size–test length combination and dimensional structure (ρ = 0.5). MELO, multidimensional Elo. Points and error bars represent means and 95% Monte Carlo confidence intervals across 500 replications. Rows correspond to the dimensional structure (between-item vs. within-item) and columns to the sample size–test length combination (*N* = 300/L = 120, *N* = 400/L = 90, *N* = 500/L = 72, and *N* = 600/L = 60). True difficulty was binned with cutpoints at −2, −1, 0, 1, and 2.

For the partial adaptive proportions, the bias patterns were intermediate and structure-dependent. In the between-item structure, increasing the adaptive selection proportion yielded a gradual transition from inward bias (at prop = 1/3 and 1/2) to outward bias (at prop = 2/3). In the within-item structure, the partial adaptive conditions largely tracked the random selection pattern, maintaining a regression-to-the-mean bias across all sample size–test length combinations.

#### 4.3.2 RMSE and bias of estimated abilities

Table 8 presents the ability estimation RMSE and Monte Carlo standard error (MCSE) aggregated across all dimensions, by sample size–test length combination, adaptive selection proportion, and dimensional structure. For both dimensional structures, ability RMSE increased as test length decreased (and sample size increased) across the four conditions. The effect of adaptive selection proportion differed markedly between structures. Under the between-item structure, RMSE followed a U-shaped pattern: relative to fully random selection, partial adaptive proportions (prop = 1/3, 1/2, 2/3) yielded modest reductions in RMSE, with the minimum occurring at the 1/2 proportion, whereas fully adaptive selection produced a substantial elevation in RMSE across all sample size–test length combinations. In contrast, under the within-item structure, RMSE decreased monotonically as the adaptive selection proportion increased; fully adaptive selection consistently produced the lowest ability RMSE, with reductions observed at each increment of adaptive proportion.

**Table 8:** Ability RMSE and Monte Carlo standard error by sample size and test length, adaptive selection proportions, and dimensional structure.

Dimensional structure	Adaptive selection proportion	*N* = 300/L = 120	*N* = 400/L = 90	*N* = 500/L = 72	*N* = 600/L = 60
Between-item	0/3 (random)	0.3654 (0.0004)	0.4111 (0.0004)	0.4484 (0.0004)	0.4802 (0.0004)
	1/3	0.3533 (0.0004)	0.3956 (0.0004)	0.4323 (0.0004)	0.4634 (0.0003)
	1/2	0.3526 (0.0004)	0.3938 (0.0004)	0.4306 (0.0004)	0.4625 (0.0004)
	2/3	0.3557 (0.0004)	0.3993 (0.0004)	0.4370 (0.0004)	0.4697 (0.0004)
	3/3 (full)	0.4762 (0.0006)	0.5374 (0.0006)	0.5749 (0.0006)	0.6008 (0.0005)
Within-item	0/3 (random)	0.3838 (0.0005)	0.4207 (0.0004)	0.4495 (0.0004)	0.4741 (0.0004)
	1/3	0.3702 (0.0004)	0.4039 (0.0004)	0.4318 (0.0004)	0.4556 (0.0004)
	1/2	0.3662 (0.0004)	0.3986 (0.0004)	0.4260 (0.0004)	0.4489 (0.0004)
	2/3	0.3630 (0.0004)	0.3941 (0.0004)	0.4204 (0.0004)	0.4433 (0.0003)
	3/3 (full)	0.3581 (0.0004)	0.3887 (0.0004)	0.4139 (0.0004)	0.4364 (0.0003)

RMSE, Root Mean Squared Error; MCSE, Monte Carlo standard error; values in parentheses are MCSEs; N, sample size; L, test length; 0/3 (random), fully random selection; 1/3, 1/2, 2/3, adaptive selection proportions; 3/3 (full), fully adaptive selection. All values are means across 500 replications.

Figure 8 presents the ability estimation RMSE of MELO conditional on true ability bins, which complements the aggregate findings in Table 8 by revealing how calibration accuracy is distributed across the ability continuum. For both dimensional structures, the incorporation of adaptive item selection attenuated the RMSE at the extremes of the ability distribution relative to fully random selection. Under the between-item structure (upper panel), fully random selection produced a U-shaped profile characterized by elevated RMSE at both tails, which became increasingly pronounced as test length decreased (and sample size increased). Partial adaptive proportions yielded flatter and lower RMSE profiles across the theta continuum, whereas fully adaptive selection produced a relatively flat but moderately elevated RMSE pattern that improved upon random selection at the tails yet was less accurate than the partial conditions in the center of the distribution. In contrast, under the within-item structure (lower panel), random selection generated a severe U-shaped pattern with dramatically inflated RMSE at the ability extremes. Increasing the adaptive selection proportion progressively flattened this profile, with fully adaptive selection achieving the most uniform accuracy across bins and substantially reducing extreme errors.

**Figure 8:**
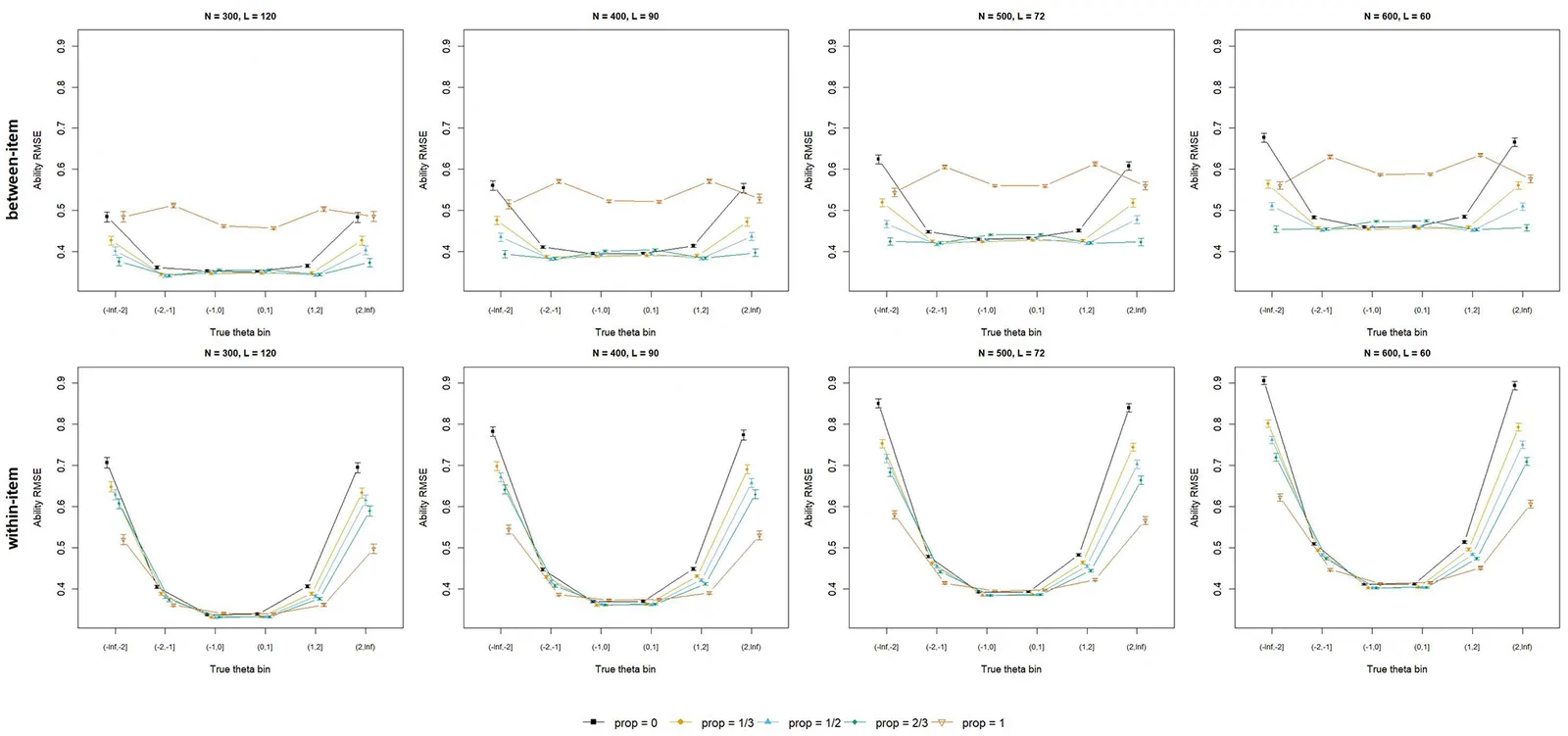
Ability RMSE of MELO conditional on true ability, comparing different adaptive selection proportions by sample size–test length combination and dimensional structure (ρ = 0.5). RMSE, root mean square error; MELO, multidimensional Elo. Points and error bars represent means and 95% Monte Carlo confidence intervals across 500 replications. Rows correspond to the dimensional structure (between-item vs. within-item) and columns to the sample size–test length combination (*N* = 300/L = 120, *N* = 400/L = 90, *N* = 500/L = 72, and *N* = 600/L = 60). True ability was binned with cutpoints at −2, −1, 0, 1, and 2.

Figure 9 presents the conditional ability estimation bias of MELO. Under the between-item structure (upper panel), partial adaptive selection proportions (prop = 1/3, 1/2, 2/3) exhibited an inward (regression-to-the-mean) pattern, wherein examinees with low true ability were positively biased and those with high true ability were negatively biased. The magnitude of this inward bias attenuated as the adaptive selection proportion increased. However, under fully adaptive selection (prop = 1), this pattern reversed dramatically, displaying a marked outward bias pattern in which low-ability examinees were substantially underestimated and high-ability examinees were overestimated. In contrast, under the within-item structure (lower panel), all adaptive selection conditions including fully adaptive consistently showed an inward bias pattern. Moreover, the magnitude of this inward bias decreased monotonically as the adaptive selection proportion increased.

**Figure 9:**
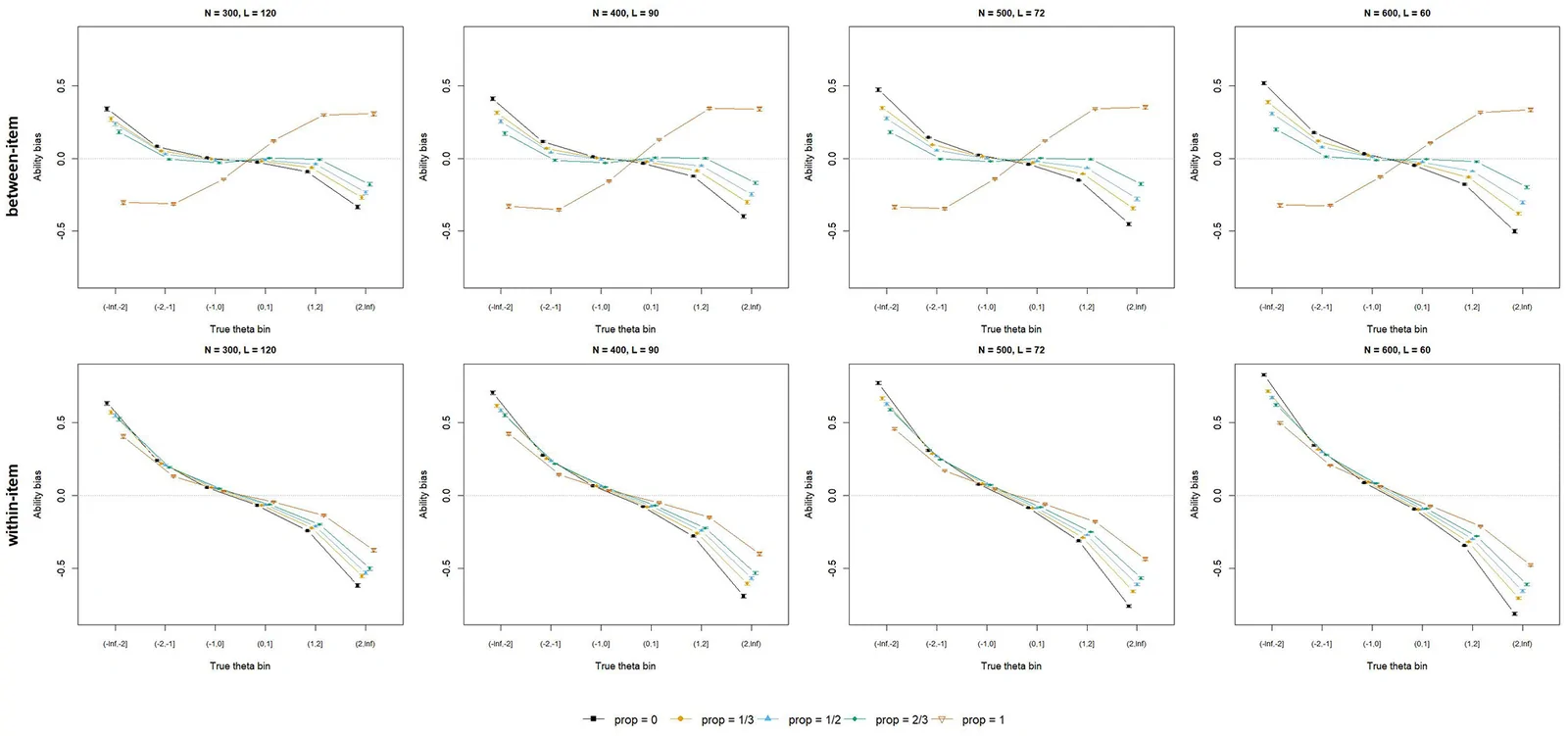
Ability bias of MELO conditional on true ability, comparing different adaptive selection proportions by sample size–test length combination and dimensional structure (ρ = 0.5). MELO, multidimensional Elo. Points and error bars represent means and 95% Monte Carlo confidence intervals across 500 replications. Rows correspond to the dimensional structure (between-item vs. within-item) and columns to the sample size–test length combination (*N* = 300/L = 120, *N* = 400/L = 90, *N* = 500/L = 72, and *N* = 600/L = 60). True ability was binned with cutpoints at −2, −1, 0, 1, and 2.

#### 4.3.3 Item exposure analysis

Table 9 presents item exposure statistics for the 180 calibrated items, including median item exposure, coefficient of variation (CV), and underexposure rate. Under fully random selection, item exposure was highly uniform across all conditions, with median values close to the expected value of 200, CVs below 0.06, and no underexposed items. As the adaptive selection proportion increased, exposure variability rose substantially in both dimensional structures, though the magnitude of this increase differed markedly between structures. In the between-item structure, the CV escalated from approximately 0.04 under random selection to 0.40–0.44 under fully adaptive selection, with the underexposure rate increasing from 0% to 12.09–15.83%. By contrast, the within-item structure exhibited more moderate exposure imbalance, with CVs reaching 0.33–0.35 and underexposure rates of 7.31–8.38% under full adaptation. These findings indicate that adaptive item selection induced greater exposure inequality in the between-item structure than in the within-item structure. Additionally, for the between-item structure under fully adaptive selection, both the CV and underexposure rate tended to increase as sample size increased and test length decreased, whereas the within-item structure showed relatively stable or slightly decreasing underexposure rates across the same conditions.

**Table 9:** Median item exposure, coefficient of variation, and underexposure rate by dimensional structure, adaptive selection proportion, and sample size–test length combination.

Dimensional structure	Adaptive proportion	*N* = 300/L = 120			*N* = 400/L = 90			*N* = 500/L = 72			*N* = 600/L = 60		
		Mdn	CV	% Under	Mdn	CV	% Under	Mdn	CV	% Under	Mdn	CV	% Under
Between-item	0/3 (random)	200.06	0.041	0.00	200.02	0.050	0.00	199.93	0.055	0.00	199.92	0.058	0.00
	1/3	205.17	0.130	0.00	204.00	0.119	0.00	201.97	0.119	0.00	200.44	0.129	0.00
	1/2	200.70	0.207	0.03	208.74	0.184	0.06	205.54	0.175	0.06	202.60	0.179	0.06
	2/3	195.58	0.286	1.99	210.91	0.256	2.82	210.64	0.242	2.75	205.83	0.240	2.76
	3/3 (full)	192.15	0.401	12.09	205.24	0.404	13.92	214.06	0.416	15.45	210.81	0.438	15.83
Within-item	0/3 (random)	200.03	0.041	0.00	200.03	0.050	0.00	200.00	0.055	0.00	199.97	0.058	0.00
	1/3	202.17	0.106	0.00	200.98	0.112	0.00	199.07	0.128	0.00	197.25	0.144	0.00
	1/2	203.07	0.168	0.02	202.12	0.155	0.06	200.57	0.168	0.06	198.15	0.189	0.08
	2/3	200.88	0.232	0.93	203.19	0.207	1.25	202.52	0.211	1.34	199.41	0.229	1.34
	3/3 (full)	202.19	0.342	8.38	205.55	0.334	7.67	201.87	0.327	7.37	201.03	0.350	7.31

Underexposed items were administered fewer than 100 times. % Under, percentage of underexposed items; CV, coefficient of variation; Mdn, median; 0/3 (random), fully random selection; 1/3, 1/2, 2/3, adaptive selection proportions; 3/3 (full), fully adaptive selection; *N*, sample size; L, test length. All values are means across 500 replications.

Correlations between item exposure and absolute item difficulty estimation error can be found in Table 10. Under fully random selection, correlations were negligible for both dimensional structures (approximately −0.02). As the adaptive selection proportion increased, correlations became progressively more negative, indicating that less frequently administered items tended to have larger absolute estimation errors. This pattern was substantially more pronounced in the between-item structure, where fully adaptive selection yielded strong negative correlations ranging from −0.6657 to −0.7069, compared with the within-item structure, which exhibited only moderate negative correlations under full adaptation (ranging from −0.1740 to −0.3047). Additionally, under fully adaptive selection, the between-item structure showed increasingly negative correlations as sample size rose and test length decreased, whereas the within-item structure displayed the opposite trend.

**Table 10:** Spearman rank-order correlations between item exposure and absolute estimation error by dimensional structure, adaptive selection proportion, and sample size–test length combination.

Dimensional structure	Adaptive proportion	*N* = 300/L=120	*N* = 400/L=90	*N* = 500/L=72	*N* = 600/L=60
Between-item	0/3 (random)	−0.0176 (0.0033)	−0.0253 (0.0033)	−0.0301 (0.0034)	−0.0253 (0.0033)
	1/3	−0.1248 (0.0035)	−0.1100 (0.0035)	−0.0833 (0.0034)	−0.0844 (0.0033)
	1/2	−0.1630 (0.0032)	−0.1620 (0.0033)	−0.1396 (0.0034)	−0.1266 (0.0034)
	2/3	−0.2424 (0.0032)	−0.2466 (0.0032)	−0.2278 (0.0034)	−0.2253 (0.0033)
	3/3 (full)	−0.6657 (0.0022)	−0.6600 (0.0022)	−0.6827 (0.0022)	−0.7069 (0.0020)
Within–item	0/3 (random)	−0.0176 (0.0034)	−0.0186 (0.0034)	−0.0201 (0.0033)	−0.0224 (0.0034)
	1/3	−0.1167 (0.0032)	−0.0992 (0.0033)	−0.0916 (0.0032)	−0.1041 (0.0032)
	1/2	−0.1325 (0.0031)	−0.1320 (0.0033)	−0.1261 (0.0032)	−0.1302 (0.0033)
	2/3	−0.1483 (0.0032)	−0.1342 (0.0033)	−0.1336 (0.0031)	−0.1467 (0.0032)
	3/3 (Full)	−0.3047 (0.0036)	−0.2597 (0.0034)	−0.1992 (0.0034)	−0.1740 (0.0033)

Values in parentheses are Monte Carlo standard errors. Absolute estimation error refers to the absolute deviation of estimated item difficulty from true item difficulty. 0/3 (random), fully random selection; 1/3, 1/2, 2/3, adaptive selection proportions; 3/3 (full), fully adaptive selection; *N*, sample size; L, test length. All correlations are means across 500 replications.

### 4.4 Real data demonstration

Table B1 in Appendix B reports item difficulty estimates from the MIRT Rasch model and the MELO algorithm for all 32 items, grouped by content domain. For the MIRT Rasch model, the resulting difficulty parameters ranged from −4.454 to 1.909 (M = −0.76, SD = 1.55). For the MELO algorithm, online joint estimation yielded difficulty parameters ranging from −3.588 to 1.949 (M = −0.61, SD = 1.38). The Pearson correlation between MIRT and MELO item difficulties was strong, *r*_(30)_ = 0.997, *p* < 0.001, indicating near-perfect correspondence. By dimension, correlations were equally strong: S1, *r*_(11)_ = 0.994; S2, *r*_(5)_ = 0.997; and S3, *r*_(10)_ = 0.998, all *p*s < 0.001.

Examinee ability estimates were recovered via expected a posteriori (EAP) scoring for MIRT and via sequential stochastic approximation for MELO. MIRT EAP scores were centered near zero across all three dimensions (M = −0.001, SD = 0.71–0.79), whereas MELO estimates exhibited small positive offsets (M = 0.08 for S1, 0.015 for S2, and 0.04 for S3), consistent with the absence of a population-mean constraint in the MELO update rule. Correlations between MIRT EAP and MELO theta estimates were strong and positive: S1, *r*_(598)_ = 0.873; S2, *r*_(598)_ = 0.817; and S3, *r*_(598)_ = 0.902, all *p*s < 0.001. These values indicate substantial convergent validity, with the S3 (Physics) dimension showing the highest correspondence and the S2 (Biology) dimension the lowest, the latter likely reflecting the smaller item pool (*n* = 7) available for online calibration.

## 5 Discussion

This study extended the Elo rating system to multidimensional tests, proposing the MELO method as an alternative to conventional likelihood-based estimation, which typically requires relatively large calibration samples and may fail to converge when data are sparse. To examine the performance of MELO in item calibration, we conducted two simulation studies representing two distinct small-sample pretest scenarios. Study 1 simulated a linear fixed-length pretest, in which all N examinees completed the full set of items. Study 2 simulated a computer-based adaptive pretest administered from an item pool; this design has the potential to improve examinees' testing experience by tailoring item difficulty to the examinee's ability level.

Study 1 showed that, under the simulated conditions, the relative estimation accuracy of MELO and MIRT depended on both the dimensional structure and the inter-dimensional correlation. For item difficulty, MIRT was consistently more accurate under the between-item structure; under the within-item structure, this advantage attenuated as the correlation increased and reversed in favor of MELO at ρ = 0.8. Much of MELO's higher overall item RMSE was concentrated in a small subset of extremely difficult items (*b* > 2), reflecting systematic inward shrinkage rather than lower precision. For ability estimation, MELO yielded slightly higher RMSE under the between-item structure; under the within-item structure, its RMSE was substantially higher at low to moderate correlations but lower than MIRT's at ρ = 0.8.

Study 2 revealed that the consequences of adaptive item selection likewise depended on the dimensional structure. Under the within-item structure, higher adaptive-selection proportions were associated with lower ability RMSE, whereas item calibration was most accurate at intermediate adaptive proportions. Under the between-item structure, partial adaptive selection produced modest gains in ability estimation, but fully adaptive selection led to substantially higher item difficulty and ability RMSE as well as increasingly unequal item exposure. Among the tested combinations with equal total response counts, configurations with fewer examinees and longer tests tended to yield lower RMSE.

### 5.1 An approach for preliminary item calibration with small samples

The MELO method proposed in this study integrates the compensatory ability assumption of multidimensional Elo extensions (Park et al., 2019) with the dynamic parameter updating approach of Elo-based calibration (Doebler et al., 2015). Prior work on Elo-type methods in educational measurement has focused primarily on ability estimation; item parameters were typically treated as known or updated only incidentally (Park et al., 2019). The present study instead targeted item calibration itself, evaluating whether MELO can recover item difficulty parameters under the small-sample conditions that characterize preliminary calibration during item pool construction.

The present findings suggest that MELO can serve as a useful alternative to likelihood-based estimation in this stage, where calibration samples are small and accumulate gradually. First, the accuracy gap between MELO and MIRT was modest in absolute terms and was largely confined to a small subset of extremely difficult items (b > 2), where MELO's higher overall RMSE originated from systematic inward bias. For the easy-to-moderate items that constitute the bulk of an item pool, the two methods differed only slightly in precision. Moreover, under the within-item structure, MIRT's advantage weakened as the inter-dimensional correlation increased, becoming statistically non-significant in some conditions and reversing in favor of MELO at *N* = 150 and ρ = 0.80.

Second, MELO never failed to produce estimates, whereas MIRT failed to converge or yield admissible estimates in a substantial proportion of replications under the high-correlation conditions (e.g., convergence rates of 25.60%−36.80% under the between-item structure at ρ = 0.80). Because MELO involves no iterative convergence procedure, it yielded estimates in all replications. This robustness is particularly relevant to preliminary calibration, in which sparse data are the rule rather than the exception.

Third, because MELO updates item and ability parameters jointly as each response arrives, it is designed for online, cold-start calibration in which data accumulate sequentially, and it can supply continuously improving starting values for subsequent formal calibration as pretest data accumulate. MELO is therefore not intended to replace likelihood-based estimation in general: once a sufficiently large calibration sample has been obtained, the accumulated data should be analyzed with MIRT or a comparable batch method.

It should be acknowledged that MELO's estimates are not unbiased. However, the conditional nature and location of this bias qualify its practical severity. Conditional bias analyses showed that item difficulty bias takes the form of systematic regression-to-the-mean shrinkage: difficulty was slightly overestimated for the easiest items, essentially unbiased for items of moderate difficulty, and increasingly underestimated for difficult items, with the bias concentrated in the extreme bin (b > 2). Ability estimates exhibited the same conditional structure, with low-ability examinees overestimated and high-ability examinees underestimated. Because this bias is monotonic in true difficulty, it preserves the rank ordering of items, a property that is most consequential for preliminary calibration purposes (such as screening items and providing starting values for subsequent formal calibration). Mechanistically, the inward pattern plausibly reflects the interplay of zero initialization and decaying learning rates: all estimates begin at the center of the scale, and item parameters with extreme true difficulties. which are fewer in number, retain a residual pull toward the initialization point. Moreover, shrinkage-type bias is well-understood in the measurement literature: it diminishes as responses accumulate, and preliminary MELO estimates are intended not as final parameters but as starting values or priors for formal calibration with larger samples, at which point the residual shrinkage is superseded.

### 5.2 Online item calibration in adaptive testing without anchor items

Computerized adaptive testing (CAT), computerized adaptive practice (CAP), intelligent tutoring systems (ITSs), and artificial intelligence (AI)-driven item generation have become integral to contemporary educational technology (e.g., Graesser et al., 2018; He and Chen, 2020). These systems personalize learning resources and test items according to students' ability levels and academic progress, thereby substantially improving both learning efficiency and instructional quality. However, all such adaptive and intelligent systems depend upon large-scale item pools, whose utility fundamentally rests on accurately estimated item parameters. Integrating item calibration directly into adaptive systems would substantially reduce calibration costs while providing examinees with informative feedback and a positive testing experience throughout the calibration process.

This idea has a well-established counterpart in the measurement literature: online calibration, in which new items are calibrated while being administered to examinees alongside operational items. However, classic online calibration methods (e.g., Chen and Wang, 2016; Chen et al., 2017; Yuan et al., 2023) presuppose a well-calibrated operational item pool with anchor items and estimate only a small number of newly administered pretest items. The scenario examined in Study 2 is fundamentally different: it is a cold-start setting in which no calibrated item pool exists, no anchor items are available, and item and ability parameters must be updated simultaneously after every response during adaptive administration. The fixed-parameter frameworks underlying existing online calibration methods are therefore not applicable. For this reason, Study 2 was designed as an internal comparison within MELO, examining how item selection adaptivity and test configuration (N × L combinations at a constant total number of responses) affect calibration accuracy, with the inter-dimensional correlation fixed at ρ = 0.50 and the average per-item exposure fixed at approximately 200 to match the per-item data volume of the *N* = 200 condition in Study 1.

The feasibility of this anchor-free design rests on the self-correcting nature of Elo's joint updating: both ability and item ratings are updated after every learner–item interaction, so inaccurate early estimates are gradually corrected as responses accumulate rather than permanently biasing item calibration (Vermeiren et al., 2026). At the same time, adaptive testing introduces a coupling between estimation and subsequent data collection: current parameter estimates guide item selection, and the selected items determine which parameters receive further updating opportunities. Because Elo updating allows both ability and item parameters to improve as responses accumulate, this coupling does not amount to permanent contamination from early estimation error; rather, in finite testing sequences, adaptive selection may alter the allocation of responses across items before all parameters have stabilized. Notably, this self-correcting property holds under random or fixed item exposure; it can break down when item selection is fully driven by the currently updated ratings themselves, in which case rating variance may inflate (Bolsinova et al., 2026). This coupling, and the conditions under which it helps or harms calibration, are the focus of Study 2.

Study 2 delineates the conditions under which adaptive item selection can benefit anchor-free online calibration with MELO. The benefits depend on the dimensional structure and the degree of adaptivity. Under the within-item structure, adaptive selection was broadly beneficial: ability estimation precision improved monotonically with the adaptive proportion, and item exposure remained acceptably balanced even under fully adaptive selection. This robustness is structural rather than incidental. The matching statistic for two-dimensional items is the sum of the measured latent traits, that is, the linear predictor of the compensatory model. Its variance, 2(1 + ρ), exceeds that of any single dimension, so items at the difficulty extremes receive informative administrations roughly five times as often as under per-dimension matching. Moreover, an examinee extreme on one dimension generates matched demand in two item pools simultaneously, so tail demand is distributed across item subsets and the minimum exposure rate of the pool is effectively raised.

Under the between-item structure, by contrast, only partial adaptivity was beneficial: a late adaptive phase following a random warm-up improved ability estimation without degrading item calibration, whereas fully adaptive selection concentrated exposures on a restricted subset of the item pool (underexposure rates of 12.09%−15.83%), left items at the difficulty extremes essentially uncalibrated near their zero starting values, and inflated conditional item RMSE 3- to 4-fold in those bins. Ability RMSE rose only modestly, because examinees were still measured with the well-calibrated region of the pool. The conditional bias patterns reinforce this interpretation (Figures 7, 9). Under random and partial-adaptive selection, item difficulty bias retained the inward, regression-to-the-mean form observed in Study 1, and its magnitude attenuated as the adaptive proportion increased, plausibly because better-targeted administrations move estimates away from their zero starting values more quickly. Under fully adaptive selection, however, this pattern reversed into a marked outward bias: easy items were substantially underestimated and difficult items overestimated, and the magnitude of the bias grew with sample size. This reversal mirrors the rating-variance inflation predicted when item selection is driven by currently updated ratings (Bolsinova et al., 2026). Once selection depends on interim estimates, mistargeted administrations push extreme parameters further from their true values instead of correcting them. Under the within-item structure, the outward shift under full adaptivity was only modest, and conditional ability bias in fact attenuated monotonically as the adaptive proportion increased, consistent with the more balanced exposure distribution documented above. For anchor-free online calibration, adaptive selection is therefore most defensible under within-item multidimensionality or as a partial, late-stage component of the test; under between-item structures, full adaptivity should not be recommended without explicit exposure control.

These bias patterns have direct operational consequences. In a deployed adaptive system, the inward bias observed under random and partial-adaptive selection would cause the system to initially present items that are slightly too difficult for lower-ability examinees and too easy for higher-ability examinees, though this distortion attenuates as calibration proceeds. More critically, the outward bias under fully adaptive between-item selection represents a self-reinforcing misalignment: as extreme items drift further from their true difficulties, the adaptive algorithm increasingly mismatches examinees to items, degrading both measurement validity and user experience. Practitioners should therefore monitor not only aggregate RMSE but also conditional bias profiles during operational calibration, treating systematic outward bias in extreme difficulty bins as a warning signal that exposure control is needed.

The sample size–test length manipulations separate two distinct sources of estimation accuracy in online calibration: the amount of data accumulated per item and the amount of information available per examinee. Under random selection, item exposure was approximately uniform, and item difficulty RMSE was virtually identical across the four configurations (approximately 0.188 under the between-item structure; Table 7), indicating that item calibration accuracy depends on per-item exposure rather than on how responses are distributed across examinees and test lengths. Ability estimation, in contrast, showed a consistent test-length effect: longer tests yielded lower ability RMSE under every condition (e.g., from 0.4802 at *N* = 600/L = 60 to 0.3654 at *N* = 300/L = 120 under the between-item structure with random selection), reflecting the accumulation of response information per examinee rather than any property of the online updating rule. Once selection became adaptive, exposure was no longer uniform, and the configuration effect interacted with the adaptivity proportion in a structure-dependent manner: under the between-item structure, fully adaptive selection degraded item calibration more severely in the short-test, large-sample configurations (item difficulty RMSE rising from 0.4216 at *N* = 300/L = 120 to 0.5620 at *N* = 600/L = 60), plausibly because short tests yield less precise interim ability estimates, which in turn misdirect adaptive selection and distort the exposure distribution, whereas under the within-item structure, item calibration accuracy was largely unaffected by configuration at moderate adaptivity proportions. It should be emphasized that, because the total number of responses and the average per-item exposure were held constant by design, these comparisons isolate the effect of test configuration at a fixed calibration cost and do not imply that sample size and test length are interchangeable in practice.

These findings carry several implications for the practice of online calibration in adaptive learning and testing systems. First, the cold-start capability of MELO permits newly developed items to enter an item pool directly, without waiting for a separately assembled pretest form or a calibrated anchor set; this can shorten the interval between item development and operational use and distribute calibration across ordinary learning or testing sessions. Second, practitioners should calibrate the degree of adaptivity to the dimensional structure of their assessments: for within-item multidimensional pools, adaptive selection can be employed relatively aggressively, as it improved ability estimation at every proportion examined and did not materially compromise item calibration; for between-item pools, adaptive selection is best introduced as a late-stage component following an initial random phase, which guarantees baseline exposure for every item before selection begins to concentrate. Third, when the total volume of calibration data is fixed, administering longer tests to fewer examinees is preferable to administering short tests to many, because the resulting gains in ability estimation accuracy benefit all subsequent parameter updates. Finally, fully adaptive selection under between-item structures should not be deployed without exposure control; monitoring item exposure and underexposure rates during calibration provides a practical safeguard against the self-reinforcing exposure imbalance observed in Study 2.

### 5.3 Limitations and future directions

Several limitations of the present study should be acknowledged. First, the response model underlying MELO assumes equal discrimination across items. This assumption is restrictive; in practice, a multidimensional 2PL formulation would afford greater flexibility by allowing items to differ in their discrimination power. Extending MELO to accommodate item-specific discrimination parameters within a multidimensional 2PL framework therefore constitutes a natural direction for future research. At the same time, the equal-discrimination assumption defines a transparent boundary for the present results, within which MELO serves as a practical starting point for online calibration.

Second, the single-pass updating design implies that early examinees' ability estimates are never revisited once the item parameters have become better calibrated. A *post-hoc* re-estimation of abilities using the final calibrated item parameters would likely improve the ability estimates of early examinees. We did not implement such a step because the focus of the present study was online item calibration rather than final ability scoring, and ability estimates were finalized in real time as each examinee completed the test, mirroring how a deployed system would operate. Nevertheless, combining online calibration with a final re-scoring pass is a natural extension. Practitioners should also be aware that early users of a deployed system experience somewhat lower estimation quality; this can be mitigated by seeding the system with pre-calibrated anchor items or warm-start parameter values rather than initializing all item difficulties at zero.

Third, the present experiments targeted the calibration of an entire item pool from scratch and did not distinguish the proportions of new vs. previously calibrated items. In operational practice, calibration more often involves a mixture of new items and an existing calibrated pool, and the balance between the two may affect both calibration accuracy and the dynamics of online updating. Simulating calibration scenarios with varying proportions of new and old items would enhance the practical guidance that future research can offer.

Finally, the present findings constitute a simulation-based proof of concept and are conditional on the response processes, item-pool structures, and parameter ranges examined in Studies 1 and 2. Although the manipulated conditions permit conclusions about relative performance within these simulations, they do not establish the operational effectiveness, actual cost efficiency, or robustness of MELO in live testing systems. Field studies using operational response data are needed before the observed patterns can support practical implementation recommendations.

### 5.4 Conclusion

This study proposed MELO, a multidimensional extension of the Elo rating system, as an approach to online item calibration under small-sample, cold-start conditions in which no calibrated item pool or anchor items are available. Across two simulation studies, MELO emerged as a viable alternative to likelihood-based estimation in the preliminary stage of item pool construction: its accuracy gap relative to MIRT was modest and largely confined to extremely difficult items, it produced admissible estimates in every replication—including high-correlation conditions under which MIRT frequently failed to converge—and it significantly outperformed MIRT under the within-item structure with strong inter-dimensional correlation. Study 2 further delineated when adaptive item selection benefits anchor-free online calibration: adaptivity was broadly beneficial under within-item multidimensionality, whereas under between-item structures it was defensible only as a partial, late-stage component of the test, with fully adaptive selection requiring exposure control to avoid self-reinforcing exposure imbalance. When the total volume of calibration data is fixed, administering longer tests to fewer examinees improves ability estimation, which in turn supports subsequent parameter updates. An application to empirical data further supported the practical viability of the method: item difficulty estimates from MELO and MIRT correlated almost perfectly, and ability estimates were strongly convergent across all three content dimensions. These findings are bounded by the simulated conditions examined, the equal-discrimination assumption, and the real-time estimation design; extensions to multidimensional 2PL frameworks, *post-hoc* re-scoring, mixtures of new and calibrated items, and field studies with operational response data are needed before the present patterns can inform routine practice.

## Data Availability

The datasets presented in this study can be found in online repositories. The names of the repository/repositories and accession number(s) can be found below: https://osf.io/rwz53?view_only=0523a2f164204238a025cba7078f65ed.
