# Supplementary material for: A multidimensional Elo method for online item calibration
Source: Front Psychol. 2026 Oct 9;17:1849155. doi: 10.3389/fpsyg.2026.1849155 (PMC13690784; doi:10.3389/fpsyg.2026.1849155)
Supplement: Supplementary file 1 [file Table_1.docx]

**Appendices**

**Appendix A. Examinee Entry-Order Analysis in Study 1**

To examine whether the order in which examinees entered the system affected ability estimation accuracy in Study 1, examinees were divided within each replication into four groups based on their position in the processing sequence. Q1 comprised the earliest approximately one-quarter of examinees processed, whereas Q4 comprised the latest approximately one-quarter. Table A1 reports the mean overall ability RMSE for the four entry-order groups across 500 replications.

**Table A1**

*Overall Ability RMSE by Examinee Entry-Order Group in Study 1*

| Dimensional Structure | N | ρ | Q1 | Q2 | Q3 | Q4 |
| --- | --- | --- | --- | --- | --- | --- |
| Between-item | 150 | 0 | 0.4550 | 0.4359 | 0.4316 | 0.4302 |
|  |  | 0.5 | 0.4417 | 0.4271 | 0.4190 | 0.4133 |
|  |  | 0.8 | 0.4343 | 0.4175 | 0.4128 | 0.4085 |
|  | 200 | 0 | 0.4543 | 0.4350 | 0.4270 | 0.4262 |
|  |  | 0.5 | 0.4368 | 0.4193 | 0.4171 | 0.4136 |
|  |  | 0.8 | 0.4309 | 0.4164 | 0.4102 | 0.4072 |
|  | 250 | 0 | 0.4467 | 0.4337 | 0.4281 | 0.4244 |
|  |  | 0.5 | 0.4351 | 0.4202 | 0.4142 | 0.4135 |
|  |  | 0.8 | 0.4279 | 0.4126 | 0.4077 | 0.4062 |
| Within-item | 150 | 0 | 0.5160 | 0.5093 | 0.5104 | 0.5092 |
|  |  | 0.5 | 0.4381 | 0.4280 | 0.4261 | 0.4263 |
|  |  | 0.8 | 0.3669 | 0.3593 | 0.3578 | 0.3564 |
|  | 200 | 0 | 0.5157 | 0.5073 | 0.5109 | 0.5051 |
|  |  | 0.5 | 0.4348 | 0.4243 | 0.4270 | 0.4264 |
|  |  | 0.8 | 0.3668 | 0.3595 | 0.3571 | 0.3562 |
|  | 250 | 0 | 0.5147 | 0.5078 | 0.5049 | 0.5074 |
|  |  | 0.5 | 0.4331 | 0.4262 | 0.4256 | 0.4274 |
|  |  | 0.8 | 0.3667 | 0.3591 | 0.3562 | 0.3572 |

*Note.* Q1 denotes the earliest approximately one-quarter of examinees processed, and Q4 denotes the latest approximately one-quarter. Values are mean overall ability RMSEs across 500 replications for each condition.

Later-processed examinees generally yielded slightly more accurate ability estimates. Under the between-item multidimensional structure, Q1 had higher overall ability RMSE than Q4 in every sample-size and correlation condition. The same general pattern was observed under the within-item multidimensional structure, although differences among the entry-order groups were smaller.

**Appendix B. Detailed Item Difficulty Estimates for the Empirical Illustration**

Table B1 reports the item difficulty estimates obtained from the MIRT Rasch model and the MELO algorithm for all 32 items in the SAT12 dataset, grouped by content domain.

**Table B1**

*Item Difficulty Parameter Estimates From MIRT Rasch and MELO Algorithms*

| Item | Dimension | MIRT b | MELO b |
| --- | --- | --- | --- |
| 7 | S1 (Chemistry) | −1.35 | −1.18 |
| 9 | S1 (Chemistry) | −2.33 | −2.15 |
| 10 | S1 (Chemistry) | 0.36 | 0.33 |
| 11 | S1 (Chemistry) | −4.45 | −3.59 |
| 13 | S1 (Chemistry) | −0.80 | −0.75 |
| 15 | S1 (Chemistry) | −1.73 | −1.65 |
| 17 | S1 (Chemistry) | −3.63 | −3.14 |
| 18 | S1 (Chemistry) | 0.70 | 0.79 |
| 21 | S1 (Chemistry) | −2.69 | −2.54 |
| 22 | S1 (Chemistry) | −3.00 | −2.76 |
| 24 | S1 (Chemistry) | −1.16 | −1.11 |
| 27 | S1 (Chemistry) | −2.10 | −1.99 |
| 31 | S1 (Chemistry) | −1.86 | −1.78 |
| 1 | S2 (Biology) | 1.07 | 0.95 |
| 3 | S2 (Biology) | 1.09 | 1.05 |
| 6 | S2 (Biology) | 1.91 | 1.84 |
| 8 | S2 (Biology) | 1.59 | 1.59 |
| 16 | S2 (Biology) | 0.40 | 0.31 |
| 29 | S2 (Biology) | 0.76 | 0.73 |
| 32 | S2 (Biology) | 1.90 | 1.95 |
| 2 | S3 (Physics) | −0.34 | −0.26 |
| 4 | S3 (Physics) | 0.57 | 0.48 |
| 5 | S3 (Physics) | −0.59 | −0.51 |
| 12 | S3 (Physics) | 0.39 | 0.40 |
| 14 | S3 (Physics) | −1.13 | −1.12 |
| 19 | S3 (Physics) | −0.24 | −0.21 |
| 20 | S3 (Physics) | −2.21 | −2.20 |
| 23 | S3 (Physics) | 0.91 | 0.84 |
| 25 | S3 (Physics) | 0.59 | 0.59 |
| 26 | S3 (Physics) | 0.17 | 0.29 |
| 28 | S3 (Physics) | −0.16 | −0.08 |
| 30 | S3 (Physics) | 0.27 | 0.31 |

*Note.* S1 = Chemistry; S2 = Biology; S3 = Physics; MIRT = multidimensional item response theory; MELO = multidimensional Elo. MIRT estimates were obtained via marginal maximum likelihood with all discrimination parameters constrained to 1.0 (Rasch model). Pearson correlations between the two sets of estimates were r(11) = .994 for S1, r(5) = .997 for S2, and r(10) = .998 for S3, all ps < .001.
